# Terbium(III)-thiacalix[4]arene nanosensor for highly sensitive intracellular monitoring of temperature changes within the 303–313 K range

**DOI:** 10.1038/s41598-020-77512-1

**Published:** 2020-11-25

**Authors:** Rustem R. Zairov, Alexey P. Dovzhenko, Anastasiia S. Sapunova, Alexandra D. Voloshina, Kirill A. Sarkanich, Amina G. Daminova, Irek R. Nizameev, Dmitry V. Lapaev, Svetlana N. Sudakova, Sergey N. Podyachev, Konstantin A. Petrov, Alberto Vomiero, Asiya R. Mustafina

**Affiliations:** 1 FRC Kazan Scientific Center, Arbuzov Institute of Organic and Physical Chemistry, Russian Academy of Sciences, 8 Arbuzov str., Kazan, Russian Federation 420088; 2 Kazan (Volga region) Federal University, 18 Kremlyovskaya str., Kazan, Russian Federation 420008; 3 Zavoisky Physical-Technical Institute, FRC Kazan Scientific Center of RAS, Sibirsky tract, 10/7, Kazan, Russian Federation 420029; 4 Division of Materials Science, Department of Engineering Sciences and Mathematics, Luleå University of Technology, 971 87 Luleå, Sweden; 5 Department of Molecular Sciences and Nanosystems, Ca’ Foscari University of Venice, Via Torino 155, 30172 Venezia-Mestre, Italy

**Keywords:** Materials chemistry, Physical chemistry, Supramolecular chemistry

## Abstract

The work introduces hydrophilic PSS-[Tb_2_(TCAn)_2_] nanoparticles to be applied as highly sensitive intracellular temperature nanosensors. The nanoparticles are synthesized by solvent-induced nanoprecipitation of [Tb_2_(TCAn)_2_] complexes (TCAn - thiacalix[4]arenes bearing different upper-rim substituents: unsubstituted TCA1, tert-buthyl-substituted TCA2, di- and tetra-brominated TCA3 and TCA4) with the use of polystyrenesulfonate (PSS) as stabilizer. The temperature responsive luminescence behavior of PSS-[Tb_2_(TCAn)_2_] within 293–333 K range in water is modulated by reversible changes derived from the back energy transfer from metal to ligand (M* → T_1_) correlating with the energy gap between the triplet levels of ligands and resonant ^5^D_4_ level of Tb^3+^ ion. The lowering of the triplet level (T_1_) energies going from TCA1 and TCA2 to their brominated counterparts TCA3 and TCA4 facilitates the back energy transfer. The highest ever reported temperature sensitivity for intracellular temperature nanosensors is obtained for PSS-[Tb_2_(TCA4)_2_] (S_I_ = 5.25% K^−1^), while PSS-[Tb_2_(TCA3)_2_] is characterized by a moderate one (S_I_ = 2.96% K^−1^). The insignificant release of toxic Tb^3+^ ions from PSS-[Tb_2_(TCAn)_2_] within heating/cooling cycle and the low cytotoxicity of the colloids point to their applicability in intracellular temperature monitoring. The cell internalization of PSS-[Tb_2_(TCAn)_2_] (n = 3, 4) marks the cell cytoplasm by green Tb^3+^-luminescence, which exhibits detectable quenching when the cell samples are heated from 303 to 313 K. The colloids hold unprecedented potential for in vivo intracellular monitoring of temperature changes induced by hyperthermia or pathological processes in narrow range of physiological temperatures.

## Introduction

Lanthanide-based nanoparticles (NPs) have gained growing attention during recent decades due to their divergent application in cellular mapping and sensing. Specific photophysical characteristics of lanthanide compounds constituting the NPs significantly differentiate the latter from polymeric organic nanoparticles and quantum dots^1–4^. Narrow intensive emission bands and long lifetimes of excited states provide a higher signal-to-noise ratio than that for the organic NPs and quantum dots. Versatility of lanthanide emitters derives from the variation of lanthanide ions, while their ligand environment enables to modify both intensity and response ability of lanthanide-centered luminescence. The temperature dependence of lanthanide-centered luminescence is the well documented basis for development of noninvasive submicrometer luminescent thermometric mapping^5–19^. Real-time in vivo and in vitro monitoring of temperature in living cells is of great impact to record temperature fluctuations during magnetic hyperthermia (MHT) or photothermal therapy (PTT), two emergent modalities for thermal treatment of cancer. The inorganic NPs doped by lanthanide ions are widely documented as nanothermometers based on up-conversion luminescence^5–12,18–21^. Mostly, they utilize the luminescence intensity ratio (LIR) approach^19^. The presence of the thermally coupled f-levels is the prerequisite for the temperature sensitivity of the inorganic NPs doped by Er^22^, Tm^18^, Nd^23^ and Ho^24^ ions, where the population distribution of thermally coupled energy levels can be re-distributed with temperature change due to Boltzmann statistics. Temperature responsive behavior of the lanthanide-based NPs is greatly affected by the energy gap between the thermally coupled f-levels of lanthanide ions. Thus, both the nature of the doped or co-doped lanthanide ions and the architecture of the lanthanide-doped NPs are the tools for tuning their temperature sensitivity^7–10,19^. However, the temperature sensitivities (S) defined as the rate of change of the thermo-sensitive parameter with temperature are typically low, usually being below 1% K^−1^ at the temperatures about 300 K for the major part of the reported lanthanide-doped inorganic NPs^7–11,18,19,25–28^.

Lanthanide complexes where lanthanide ions are coordinated by organic ligands of different structure represent promising basis for thermo-responsive nanomaterial because of the presence of different mechanisms, with respect to those regulating the thermo-responsive behavior of lanthanide ions in the inorganic matrices^29^. Greater temperature sensitivities of the molecular complexes, reported as up to 4.9% K^−1^, make them very promising basis for development of the lanthanide-based thermometers^30–34^. It is worth noting that both encapsulation of lanthanide complexes and their deposition onto polymeric nanomaterial (commonly silica, due to its transparency) induce some changes in the inner-sphere environment of lanthanide ions, which, in turn, affect the thermal behavior of the complexes. However, the effects of the nanoparticulate hosts on the temperature responsive behavior of the lanthanide complexes are insufficiently studied. Moreover, the ligand environment of the ions commonly applied for these purposes (Tb^3+^ and Eu^3+^) is restricted to 1,3-diketonates. This mainly derives from both good luminescence and convenient thermal behavior of the lanthanide diketonates. However, the low solubility of the complexes in specific solvents (water or alcohol) affects the efficiency of the doping procedure restricting their use as molecular blocks of silica nanoparticles^35,36^.

The luminescent nanoparticles with core–shell morphology, where the core results from the solvent-induced nanoprecipitation of lanthanide complexes, while the hydrophilic shell derives from the deposition of the polyelectrolytes, represent very promising alternative to the polymeric nanoparticles doped by the lanthanide complexes^4,6^. The main advantage of this morphology is the facile and easy way of conversion of the molecular lanthanide complexes into the aqueous colloids. Moreover, any complexes, which are soluble in any water-miscible organic solvent, but insoluble in water, provide a basis for the transition from molecular to nanoparticulate form. However, this molecule-to-nano transformation can affect both inner- and outer-sphere environment of the terbium ions, which, in turn, changes the Tb^3+^-centered luminescence and its thermo-sensitivity.

**Figure 1:**
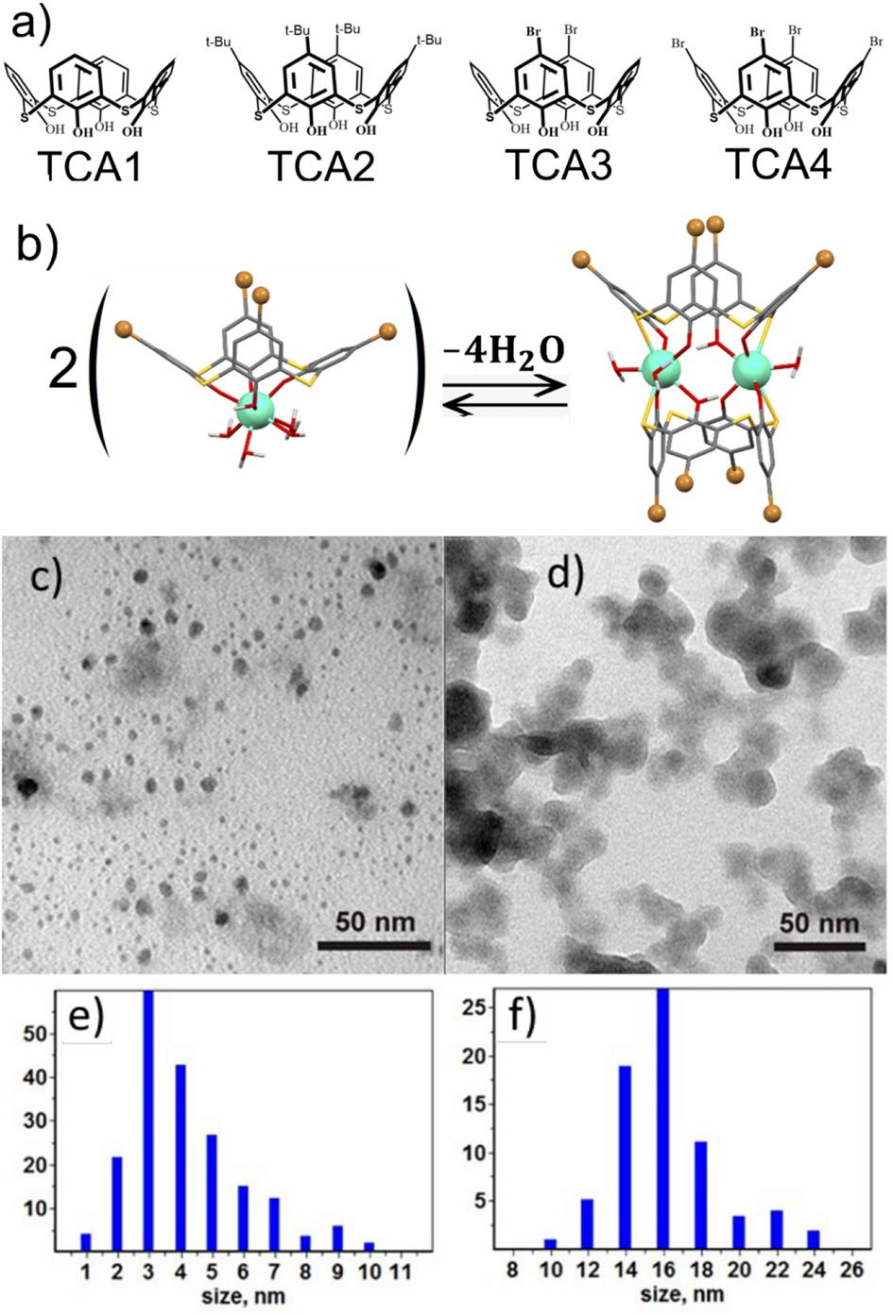
(**a**) Structures of thiacalix[4]arene ligands TCAn (n = 1–4). (**b**) Schematic illustration of equilibrium between 2:2 and 1:1 exemplified for Tb^3+^ complex with the TCA4. TEM images of dried (**c**) PSS-[Tb_2_(TCA3)_2_] and (**d**) PSS-[Tb_2_(TCA4)_2_]. (**e**,**f**) Corresponding size distribution diagrams.

Thus, the factors such as hydration, enhanced surface activity, chemical and aggregation stability and their effect on luminescence properties and thermo-sensitivity are the focus of the present report. Careful monitoring of these factors, in turn, is a prerequisite for design of novel nanoparticles with high thermo-sensitivity and good cellular uptake behavior, able to allow the luminescence response monitored in the intracellular space on the gentle heating of the cell samples. In this regard, calix[4]arene, calix[4]resorcinarene and thiacalix[4]arene derivatives provide the optimal ligand environment of Tb^3+^ ions for both efficient Tb^3+^-centered luminescence and facile conversion into the aqueous luminescent colloids with high colloid stability^4,37^. The documented colloids were revealed as efficient fluorescent contrast agents with good cellular uptake behavior^4^. Moreover, some thiacalix[4]arene derivatives has been recently reported^38,39^ as the appropriate ligand environment of Tb^3+^ ions to generate the Tb^3+^-centered luminescence exhibiting the temperature responsive behavior in physiological temperature range. In particular, the dimeric 2:2 terbium complexes with the bromo-substituted thiacalix[4]arenes provide a promising basis for design of a temperature-sensitive nanomaterial^39^. This is the reason for focusing on the optimal conditions of an efficient conversion of the complexes from molecular blocks in the organic solutions to the water dispersed hydrophilic nanoparticles. The identification of main factors, including the structure of the ligands, influencing the temperature responsivity of the Tb^3+^-centered luminescence of the aqueous colloids based on the terbium complexes with four thiacalix[4]arene derivatives, will be discussed. Thus, the four thiacalix[4]arene derivatives bearing at the upper rims different substituents, including bromines (Fig. 1a) are represented herein as the ligands.

## Experimental section

### Materials

Terbium(III) nitrate hexahydrate (Tb(NO_3_)_3_·6H_2_O, Alfa Aesar, 99.9%), gadolinium(III) nitrate hexahydrate (Gd(NO_3_)_3_·6H_2_O, Sigma-Aldrich, 99.9%), triethylamine (TEA, Acros Organics, 99%), D_2_O (Sigma , poly(sodium 4-styrenesulfonate) (PSS) (MW_avg_ = 70,000, Acros Organics), DAPI (Sigma) and sodium chloride (Sigma-Aldrich) were used as received. N,N-Dimethylformamide (DMF) (Acros Organics) was twice distilled over P_2_O_5_. Thiacalixarenes TCA1^40^, TCA2^41^, TCA3^39^ and TCA4^42^ were synthesized according to published procedures. Both ^1^H NMR spectra of the thiacalix[4]arenes and their elemental analysis data (Fig. S1 in Supplementary Material) are in good agreement with the previously reported results^39–42^.

### Synthesis of the colloids

The synthetic procedure is based on the formation of nanosized cores derived from precipitation of water insoluble Tb(III) complexes with ligands TCAn (n = 1–4) with the following adsorption of polystyrenesulfonate (PSS) molecules on their surfaces. The precipitation is induced by the pouring of 0.5 ml of the complex DMF solution under intensive stirring (2200 rpm) 2.5 ml of PSS-containing aqueous solution (1 g/L in 0.5 M solution of NaCl, pH 6.0). The complex formation in the DMF solution was achieved by mixing of the Tb(NO_3_)_3_·6H_2_O with TCAn in the 1:1 ratio, and the solution was basified by the excess amounts of triethylamine (TEA) taken in 1:8 (TCAn:TEA) ratio^43^. The resulted colloidal solution was sonicated for 30 min (sonication bath temperature was controlled to be 20 ± 2 °C). Excess amounts of PSS were separated from colloids via centrifugation (15,000 rpm, 15 min) and supernatant drain. One layered PSS-coated aqueous colloids were obtained via their redispersion (using sonication) in bidistilled water (equal to supernatant volume).

The thermo-behavior of PSS-[Tb_2_(TCAn)_2_] (n = 1–4) was examined by measuring of Tb^3+^-centered luminescence in the aerated aqueous colloids at different temperatures. The temperature acquisition performed by the water circulation thermostat required 5 min.

**Scheme 1:**
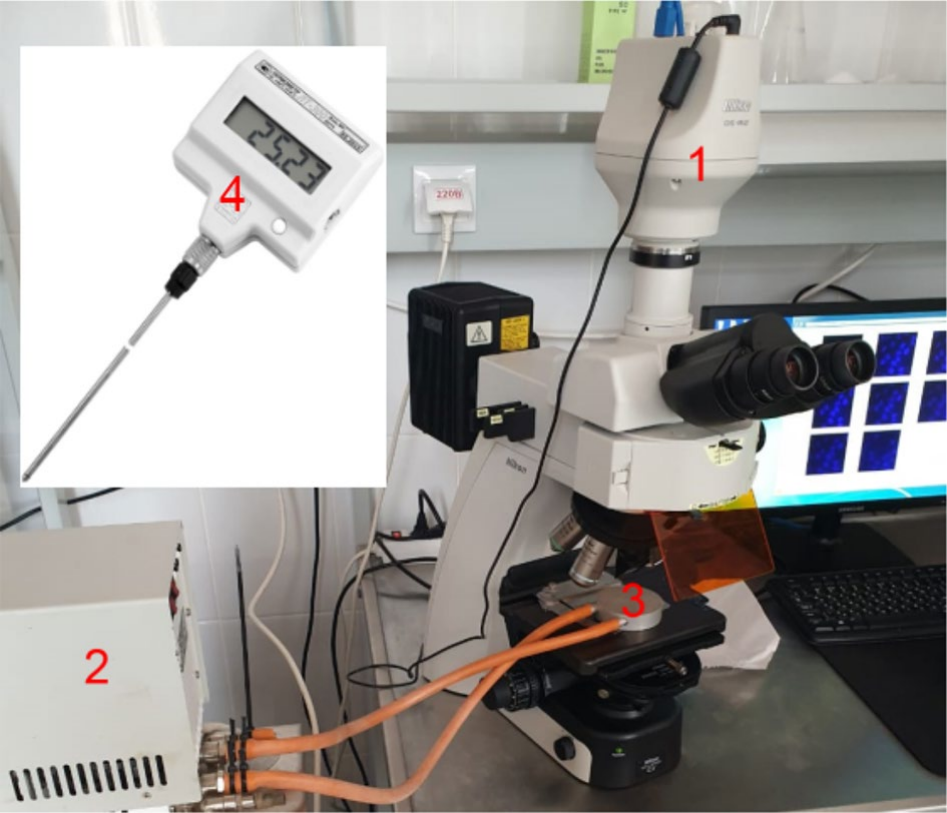
Experimental apparatus for the fluorescent microscopy measurements at different temperatures.

The monitoring of the luminescence intensity and fluorescent microscopy imaging of M-Hela cells stained by PSS-[Tb_2_(TCAn)_2_] (n = 3, 4) at different temperatures was performed in the manner shown in Scheme 1 with the use of the equipment designated in the Scheme 1 by the numbers: (1) fluorescent microscope Nikon Eclipse Ci-S fluorescence microscope (Nikon, Japan), objective CFI Plan Fluor 40X; (2) water circulation thermostat BT-20 used to establish and maintain required temperature in external contour; (3) metallic heater placed on the cover glass with the cell specimen to provide the effective heat-exchange; (4) TERMEX electronic laboratory thermometer LT-300-N (4) (measuring range − 50… + 300 °C, up to + 200 °C resolution 0.01 °C, error ± 0.05 °C) set in contact to the heater (3) for exact temperature measurements after equilibrium time of 5 min at each temperature step.

Conventional methods, such as *dynamic light scattering (DLS), transmission electron microscopy (TEM), EDS, luminescence spectroscopy and lifetime measurements, cytotoxicity assay, flow cytometry assay, fluorescence microscopy, confocal laser scanning microscopy* are described in details in Electronic supplementary material file.

## Results and discussion

**Figure 2:**
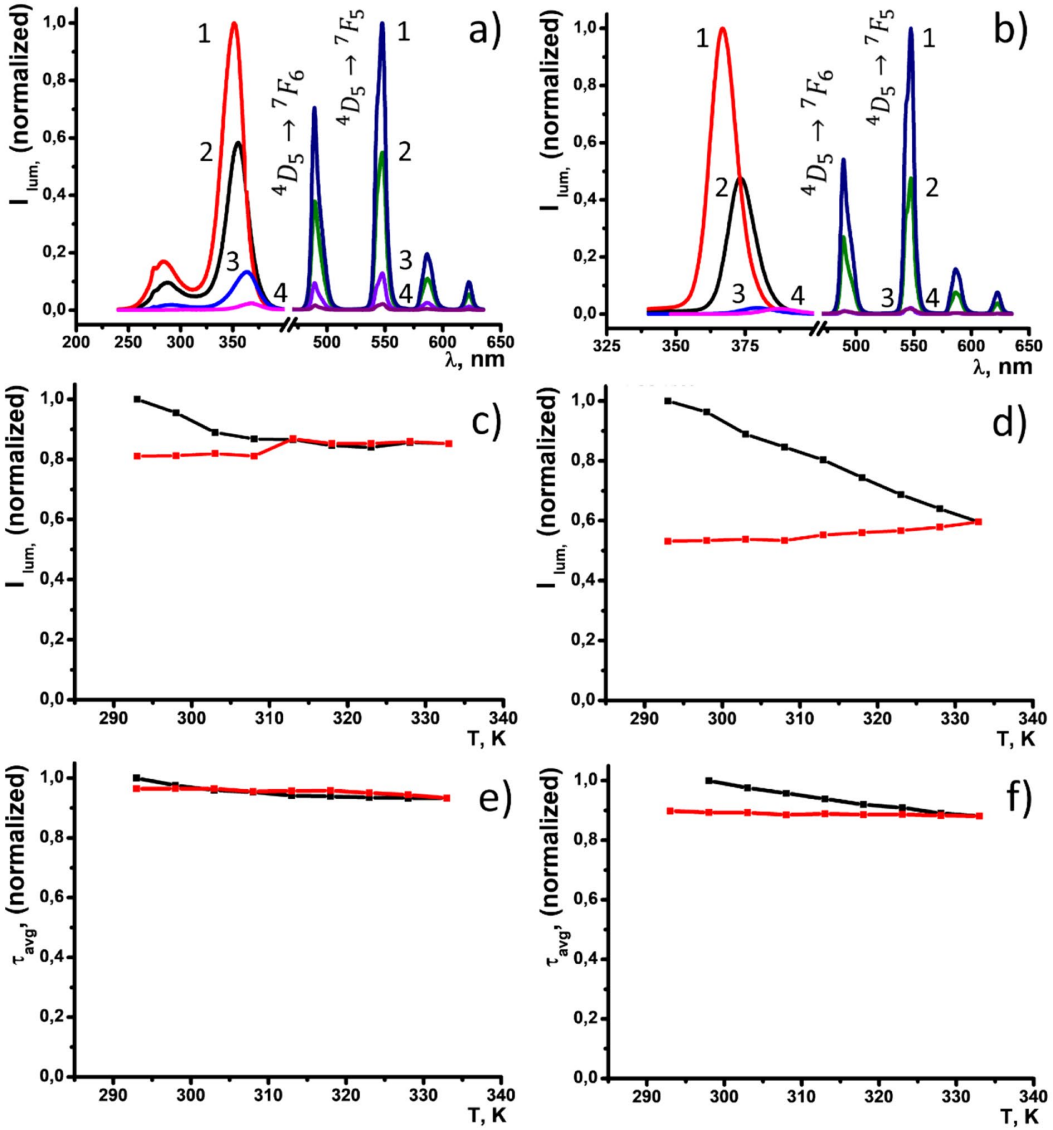
(**a**) Excitation and luminescence spectra of PSS-[Tb_2_(TCAn)_2_] ((λ_ex_, (nm (n)) = 351 (1), 355 (2), 363 (2) and 367 (4)) nanoparticles in H_2_O and (**b**) [Tb_2_(TCAn)_2_] (λ_ex_, (nm (n)) = 367 (1), 373 (2), 380 (3) and 388 nm (4)) in DMF. C = 0.75 mM for all of the samples. Normalized intensity of the luminescence band at 545 nm (**c**,**d**) and excited state lifetimes (**e**,**f**) of PSS-[Tb_2_(TCA1)_2_] (**c**,**e**) and PSS-[Tb_2_(TCA2)_2_] (**d**,**f**) in water monitored within the range of temperatures 293–333 K upon heating (black) and cooling (red).

Figure 1a illustrates the structure of four thiacalix[4]arene ligands TCAn (n = 1–4) applied in the present work. As it has been recently reported^38,39^ ligands TCAn (n = 2–4) form terbium complexes in the TEA-basified DMF solutions with the 2:2 stoichiometry derived from the sandwich-like coordination of two Tb^3+^ ions between two phenolic/phenolate rims schematically represented in Fig. 1b. It is worth noting that the 2:2 complex results from the dimerization of the 1:1 complexes, thus, the 2:2 and 1:1 complexes are equilibrated in the solutions (Fig. 2b). Both 1:1 and 2:2 complexes are electroneutral, which is the factor facilitating their nanoprecipitation when solvent is changed from DMF to water, while 2:2 complexes are thermodynamically favored versus 1:1 complex formation^38,39^. The tert-buthyl-substituted thiacalix[4]arene derivative (TCA2) has been also introduced in the present work to vary hydrophobicity of calixarene derivatives upon addition of tert-butyl groups to facilitate the solvent-induced aggregation of their complexes and to compare with TCA1.

UV–Vis study of the complex formation of TCA1 with Tb^3+^ ions in the DMF solutions evidences great similarity with TCA2. The steady state and time-resolved Tb^3+^-centered luminescence of the terbium complex with TCA1 in DMF reveals the insignificant deviation from the 2:2 complex with TCA2 (Fig. S2). The energy of the lowest triplet state (*T*_1_) of TCA2 was evaluated by the analysis of the fluorescence and phosphorescence spectra of the isostructural Gd^3+^ complexes in DMF (Fig. S2). It is worth noting that both triplet level energy of TCA2 and the average lifetime value of the Tb^3+^-centered excited state (417 nm or 23,981 cm^−1^ and 961 µs) are very close to the values recently reported for TCA1 (417 nm or 23,981 cm^−1^ and 1090 µs)^39^. This allowed to assume that the sandwich-like structure of [Tb_2_(TCAn)_2_] complexes shown in Fig. 1b is predominant for the nanoprecipitated terbium complexes in PSS-[Tb_2_(TCAn)_2_] colloids.

The terbium complexes were successfully converted into the aqueous colloids in accordance with the procedure previously optimized for the gadolinium complexes with thiacalix[4]arene derivatives. The latter is based on the aggregation process triggered by the solvent exchange from the DMF to aqueous-DMF solution of PSS with small volume percentage of DMF (for more details see the "Experimental section"). It is worth noting that the solvent-induced aggregation of the complexes is based on their insolubility in water, while the uncomplexed deprotonated ligand, Tb^3+^ ions and the excess of TEA tend to stay in the aqueous DMF solution due to their better solubility in water. The aqueous colloids resulting from the aggregation can be easily separated from the supernatant by centrifugation. The facile phase separation of the produced colloids provides the way to wash and analyze the Tb^3+^ in the supernatants. This enables to dispose of any water-soluble admixtures and to evaluate the extent of the Tb^3+^ complexes conversion from the molecular to the nanoparticulate state. The conversion extent was determined by spectrophotometric analysis of the Tb^3+^ ions in the supernatants, produced by the first phase separation after the synthesis, and the second one performed within the washing procedure. The percentages of the Tb^3+^ losses during the synthesis and the washing step are collected in Table S1. The losses of the Tb^3+^ ions during the synthetic procedure are about 3% relative to the initial concentration of the Tb^3+^ ions in the synthetic mixture, which indicates that the major extent of the complexes is converted from the molecular to the nanoparticulate state. Moreover, the EDS spectrum of the dried PSS-[Tb_2_(TCA3)_2_] colloids (Fig. S3) confirms the similar composition of the molecular and nanoparticulate states by the presence of the band (Ma = 1.240 keV) peculiar for Tb^3+^ ions and the C, O and S bands of TCA3 and PSS. The Cl band derives from the residual sodium chloride within the PSS-layer (see the synthetic procedure of the PSS-based colloids in the Experimental Section). The copper constituting of the grid is manifested by the Cu band in the spectrum (Fig. S3), while the aluminum band originating from the grid holder was subtracted from the spectrum. Unfortunately, Br peak expected in the spectrum at 1.5 keV is overlapped by dominating Al band.

The Tb^3+^ losses are lower during the washing of PSS-[Tb_2_(TCAn)_2_], being about 1.7% for TCA1, while less than 1% for TCA2-4.

Similar to PSS-[Gd_2_(TCAn)_2_] (n = 1–3)^43^, the electrokinetic potential values measured in the aqueous colloids range from − 46 to − 68 mV (Table 1), which explains their stability to aggregation manifested by the small polydispersity index and the average size below 100 nm. The TEM images of the dried colloids (Fig. 1c,d) reveal the size values of the [Tb_2_(TCAn)_2_]-based cores, which are smaller than those measured by the DLS technique according to corresponding size distribution diagrams (Fig. 1e,f). Both the thickness of the hydrated exterior polyelectrolyte layer of PSS-[Tb_2_(TCAn)_2_] colloids and their aggregation behavior are the well-known reasons for the deviation in the size-values measured by the different techniques^44^.

**Table 1:** The average sizes (d), electrokinetic potential values (ζ), polydispersity indices (PDI) evaluated from the DLS measurements of PSS-[Tb_2_(TCAn)_2_] (0.75 mM).

PSS-[Tb_2_( TCAn)_2_]	d, nm	PDI	ζ, mV	τ_avg_
L = TCA1	100.1	0.217	− 59.3 ± 1.41	0.87
L = TCA2	82.75	0.239	− 67.7 ± 1.24	0.70
L = TCA3	81.17	0.191	− 57.4 ± 1.40	0.67
L = TCA4	124.5	0.124	− 45.8 ± 1.65	0.82

### The Tb^3+^-centered luminescence of the colloids

The steady state luminescence of PSS-[Tb_2_(TCAn)_2_] colloids is manifested by four narrow bands peculiar for Tb^3+^-centered luminescence (Fig. 2a,b). Similar to [Tb_2_(TCAn)_2_] complexes in the DMF solutions^39^, the luminescence intensity is the highest for the thiacalix[4]arenes TCA1 and TCA2 compared to the bromo-substituted ligands TCA3 and TCA4. The integral intensities of the four emission bands measured in PSS-[Tb_2_(TCAn)_2_] colloids in the same concentration and instrumental conditions presented in Table 2 confirm the aforesaid tendency. The intensity ratios of the bands at 489 nm (^5^D_4_ → ^7^F_6_) and 543 nm (^5^D_4_ → ^7^F_5_) are about 0.7 for the PSS-[Tb_2_(TCAn)_2_] colloids, which is greater than the ratios 0.56–0.59 previously reported for [Tb_2_(TCAn)_2_] in the DMF solutions^39^. Since the ratio is commonly considered as the factor affected by changes in a ligand environment of terbium ions^45^, the observed difference can be explained by the hydration effect arisen from the solvent exchange on going from the DMF to the aqueous solutions.

**Table 2:** The integral intensities (I_int_) evaluated for the four emission bands in the range 470–635 nm, average lifetime values (τ_avg_) measured in PSS-[Tb_2_(TCAn)_2_] (0.75 mM) along with the τ_avg_ values for the complexes in the DMF solutions presented in brackets, and the hydration numbers (q) calculated from the luminescence measurements.

PSS-[Tb_2_(TCAn)_2_]	I_int_	τ_avg_	τ_avg_ deviation	q
n = 1	858,667	1.14 (0.96)	0.0059	1.65
n = 2	500,269	0.76 (1.09*)	0.0043	2.15
n = 3	113,577	0.77 (1.12*)	0.0024	1.93
n = 4	2696,5	0.48 (0.98*)	0.0104	3.01

*- τ_avg_ values in DMF^39^.

The sandwich-like coordination mode of the Tb^3+^ ions in [Tb_2_(TCAn)_2_] shown in Fig. 1b leaves only two water molecules in the inner-sphere of the metal ions^38^. Thus, the measurements of hydration numbers (q) of the Tb^3+^ ions in PSS-[Tb_2_(TCAn)_2_] were performed for confirming the complex stoichiometry. For this reason, in accordance with the well-known procedure^44,46^ the excited state lifetimes were measured for PSS-[Tb_2_(TCAn)_2_] in both H_2_O and D_2_O and reported in Table S2. Comparison of the excited state lifetimes of Ln(III) in specific complexes^46^ and colloidal particles^44^ in H_2_O and D_2_O solutions allows calculating the number of coordinated water molecules with the uncertainty in q (number of water molecules) of ± 0.5 by the use of the Horrocks equation (Eq. 1).

1 $${\text{q}}_{{{\text{Ln}}}} = {\text{ A}}_{{{\text{Ln}}}} (\tau^{{ - {1}}}_{{{\text{H2O}}}} - \tau^{{ - {1}}}_{{{\text{D2O}}}} )$$

The quantitative analysis of the time-resolved Tb^3+^-centered luminescence reveals the double-exponential nature of the radiation intensity decay, and the existence of two characteristic lifetime τ-values (Table S2). It is worth assuming that the longer and shorter τ values (Table S2) correspond to the complex forms with less and more hydrated Tb^3+^ ions. For comparative analysis, the average lifetime values (τ_avg_) were calculated according to the equation presented in SI along with the hydration numbers (q) presented in Table 2. The data in Table 2 reveal no significant difference in the τ_avg_ values among the ligands TCAn (n = 1–3). For comparison, the average values of the complexes in DMF are reported in brackets along with those measured in the colloids. The comparison of the lifetime values measured in PSS-[Tb_2_(TCAn)_2_] when n = 2, 3 indicates that they are smaller than those measured for TCA1 or previously reported for TCAn (n = 2–4) in the DMF solutions of [Tb_2_(TCAn)_2_] (Table 2)^38^. The tendency agrees well with the above mentioned assumption about the hydration of the complexes in the aqueous colloids. Indeed, the hydration numbers are about 2 for PSS-[Tb_2_(TCAn)_2_] (n = 1–3). Thus, the sandwich-like coordination of Tb^3+^ ions leading to the 2:2 stoichiometry is predominant in these colloids, while the small extent of the monomeric complex forms with the greater hydration number of Tb^3+^ ions (q = 5)^39^ cannot be excluded because of the shorter τ values contributing to τ_avg_ (Table S2).

The lower average τ-value of PSS-[Tb_2_(TCAn)_2_] for TCA4 correlates with the hydration number equal to 3. The greater hydration number revealed in PSS-[Tb_2_(TCA4)_2_] versus PSS-[Tb_2_(TCAn)_2_] (n = 1–3) can be explained by the increased contribution of the more hydrated monomeric form.

### The thermo-behavior of the PSS-[Tb_2_(TCAn)_2_] colloids

The analysis of the temperature-induced changes in Tb^3+^-centered luminescence for PSS-[Tb_2_(TCAn)_2_] is worth preceding by discussion of the main factors responsible for the temperature dependent luminescence of [Tb_2_(TCAn)_2_] in the solutions. As it has been previously documented^39^ the triplet energy level of the ligands is of the greatest impact on the thermo-behavior of [Tb_2_(TCAn)_2_] in solution. The recently reported invariance of the Tb^3+^-centered luminescence of complex [Tb_2_(TCA2)_2_] in solution upon heating within the range 293–323 K^39^ results from the higher energy of the triplet levels relative to the resonant ^5^D_4_ level of Tb^3+^ ion (~ 20,500 cm^−1^). The difference between the energies of these levels in accordance with the Latva rule is enough to provide efficient ligand-to-metal energy transfer and insignificant energy back transfer^47,48^. The similarity in the triplet levels for TCA1 and TCA2 in the terbium complexes is the reason for the insignificant change in the luminescence of [Tb_2_(TCAn)_2_] (n = 1,2) in the solutions within the temperature range 293–323 K. However, the relative intensity of the main band at 545 nm of PSS-[Tb_2_(TCAn)_2_] (n = 1, 2) has a thermal behavior, manifested by the quenching under the heating to 333 K, which is most pronounced for TCA2 (Fig. 2c,d). The quenching keeps effective even after cooling to 293 K, thus, pointing to some chemical or photochemical degradation of the complexes in the aqueous colloids inducing irreversible quenching.

The degradation of the colloids can be monitored by measuring the Tb^3+^ ions released from the colloids to aqueous solution after the heating–cooling cycle. However, the spectrophotometric analysis of the aqueous phase separated from the colloids by centrifugation reveals the Tb^3+^ losses after the heating–cooling cycle at the level of 1–1.89% relative to the concentration of the Tb^3+^ ions in the colloids (Table S1). This value cannot explain the significant quenching (20–40% relative to the initial intensity) observed under the heating of PSS-[Tb_2_(TCAn)_2_] (n = 1, 2).

It is worth assuming a photobleaching of the colloids as a factor contributing to the observed quenching, since photo-oxidation of phenol derivatives^49,50^ can facilitate the degradation of the complexes during the luminescence measurements at different temperatures, where the samples are exposed to the irradiation for a long time (about twenty-thirty minutes). However, PSS-[Tb_2_(TCAn)_2_] (n = 1, 2) should be irradiated within two hours at least for the degradation of the luminescence on 20–30% relative to the initial value, which is much longer than the irradiation time for the heating–cooling cycle. The DLS measurements of the colloids after the heating–cooling cycle confirm the insignificant degradation of the colloids (Table S3). The time-resolved luminescence measurements indicate that the τ_avg_ values also tend to decrease after heating and similar as in the steady state luminescence the changes are irreversible: no recovery of the τ_avg_-values is observed after cooling of the heated solution (Fig. 2e,f). The irreversibility of the changes in both steady state and time-resolved luminescence points to the irreversible transformation of the colloids under heating.

**Figure 3:**
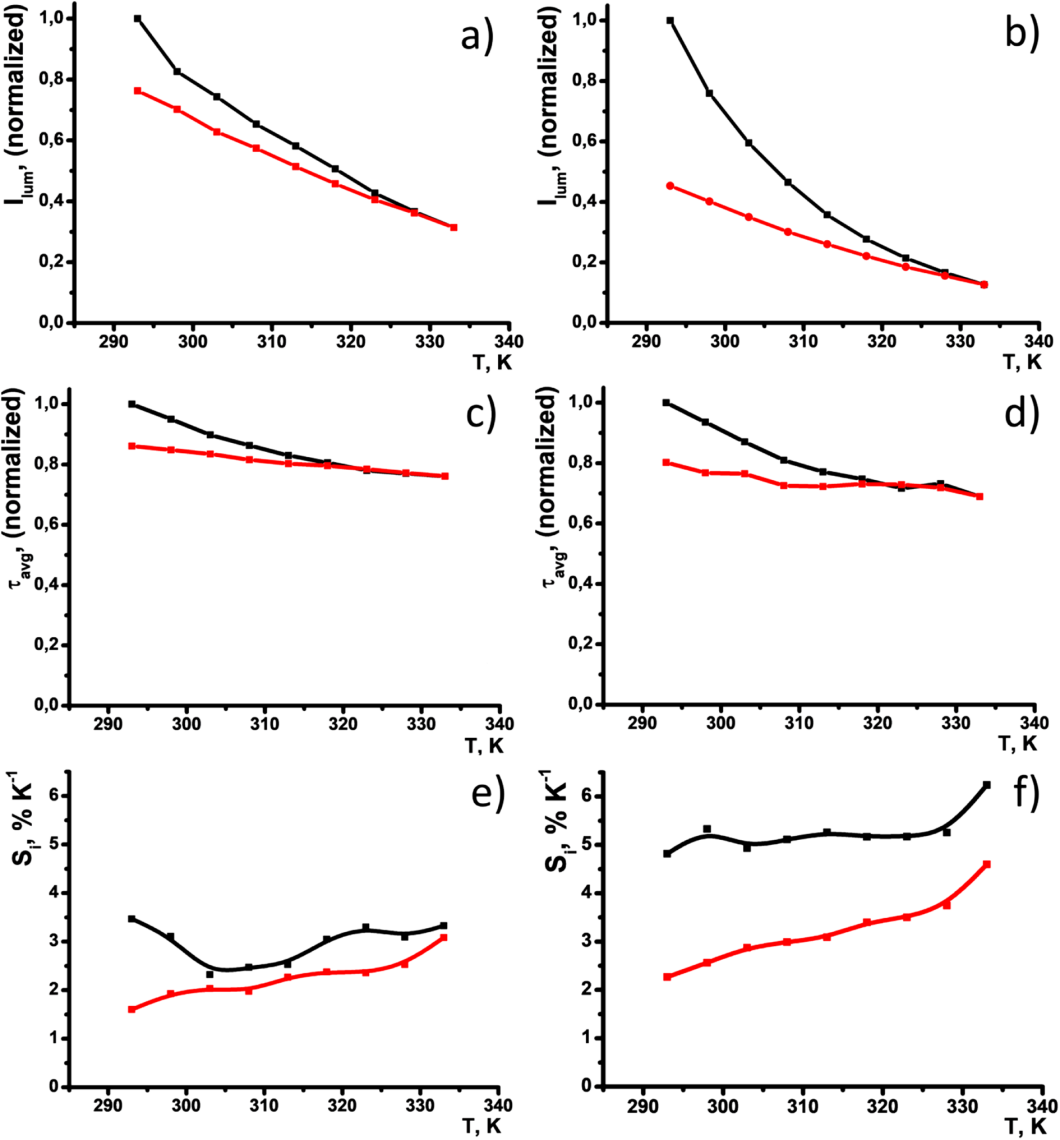
Normalized intensity of the luminescence band at 545 nm (**a**,**b**) and excited state lifetimes (**c**,**d**) of PSS-[Tb_2_(TCA3)_2_] (**a**,**c**)) and PSS-[Tb_2_(TCA4)_2_] (**b**,**d**) monitored within the range of temperatures 293–333 K upon heating (black) and cooling (red). S_I_ values at different temperatures calculated from the luminescence intensity data for PSS-[Tb_2_(TCA3)_2_] (**e**) and PSS-[Tb_2_(TCA4)_2_] (**f**).

The heating of PSS-[Tb_2_(TCAn)_2_] when n = 3, 4 results in more pronounced quenching under the heating from 293 to 333 K (Fig. 3a,b) versus the quenching of the colloids based on TCA1 and TCA2.

The luminescence of PSS-[Tb_2_(TCAn)_2_] (n = 3, 4) being quenched by the heating to 333 K tends to recover after the cooling to 293 K, although the recovery is incomplete. It is worth noting that the previously reported energies of the triplet levels for ligands TCA3 (440 nm) and TCA4 (458 nm) reveal them as much worse antenna of Tb^3+^-centered luminescence than TCA1 and TCA2 due to the greater back energy transfer from the excited level of Tb^3+^ to the triplet level of the ligand^39^. The lifetime values of PSS-[Tb_2_(TCAn)_2_] (n = 3, 4) tend to decrease by 20% relative to the initial value under the same heating. The thermo-induced decrease in the lifetime values (Fig. 3c,d) is less pronounced than that in the luminescence intensities (Fig. 3a,b). Thus, thermo-enhanced vibrational moves of the water molecules in the inner-sphere of the Tb^3+^ ions provide the heating-triggered quenching of the luminescence in smaller extent compared to back energy transfer. As it has been above mentioned the energies of the triplet states for TCA3 and TCA4 are the prerequisite for efficient energy back transfer, which is the reason for quenching of the Tb^3+^-centered luminescence. The quenching, in turn, tends to increase under the heating due to the temperature-dependent Boltzmann distribution, although the heating provides greater luminescence quenching in PSS-[Tb_2_(TCAn)_2_] (n = 3, 4) colloids than for the complexes in DMF solutions. However, the data in Fig. 3a,b reveal detectable deviation between the heating and cooling curves for ligand TCA3, which becomes greater for TCA4. It is worth assuming that different contribution of the temperature or photo-induced degradation processes to the temperature-induced quenching is the reason for the different thermal behavior of PSS-[Tb_2_(TCAn)_2_] between n = 3 and 4.

The Tb^3+^-losses after the heating–cooling cycle, being no more than 3.5% relative to the initial concentration of PSS-[Tb_2_(TCAn)_2_] (n = 3, 4), indicates rather poor degradation of the complexes under their treatment within the cycle (Table S1). However, the photobleaching of PSS-[Tb_2_(TCAn)_2_] becomes greater going from ligands TCA1 and TCA2 to the bromo-substituted ligands TCA3 and TCA4 (Fig. S4). Moreover, the photobleaching of PSS-[Tb_2_(TCAn)_2_] tends to be larger with the increase of the number of the bromo-substituents of the ligand. The photobleaching explains the degradation of Tb^3+^-centered luminescence after the heating–cooling cycle (Fig. 3a,b) on 23.7% and 54.7% relative to the initial intensity for PSS-[Tb_2_(TCA3)_2_] and PSS-[Tb_2_(TCA4)_2_], respectively. Indeed, the aforesaid percentages of the degradation agree with the real time duration (about 20–25 min for more details see the Exp. Section) required for the luminescence measurements during the heating–cooling cycle within 293–333 K. It is worth noting that both nanoparticulate state and aqueous environment are the factors enhancing the photobleaching, since the degradation of [Tb_2_(TCA4)_2_] in the DMF solutions after continuous irradiation for a half of an hour is no more than 10% of the initial luminescence intensity^39^. The literature data on the photo-oxidation of the phenols enhanced by their chloro-substitution^50^ can be assumed as an explanation of the bromo-substitution effect on the photobleaching. Thus, the upper-rim bromination of the thiacalix[4]arene ligands is the reason for the partially-reversible temperature-induced quenching of PSS-[Tb_2_(TCA3)_2_] and PSS-[Tb_2_(TCA4)_2_] through the back energy transfer mechanism, although the increased photobleaching contributes to the quenching of the colloids reducing the reversibility of their thermal behavior.

**Figure 4:**
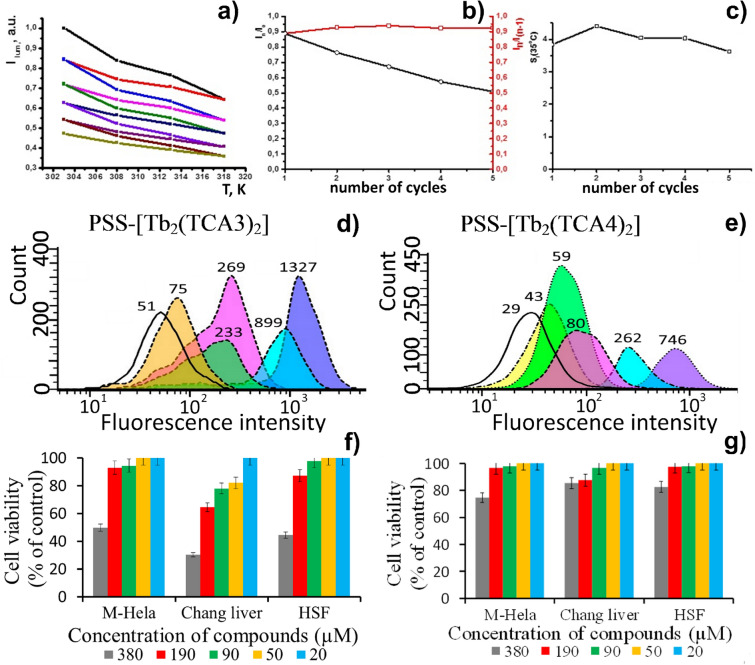
(**a**) Five “heating–cooling” cycles for PSS-[Tb_2_(TCA3)_2_] within the range of human physiological temperatures. (**b**) I_n_/I_0_ and I_n_/I_(n-1)_ values versus the number of the cycles (n). (**c**) *S*_*I*_-values at 308 K versus n. Flow cytometry cellular uptake study of (**d**) PSS-[Tb_2_(TCA3)_2_] and (**e**) PSS-[Tb_2_(TCA4)_2_]. Each peak designated with corresponding luminescence intensity values. The viability of human cell line M-Hela, Chang liver and HSF under the action of PSS-[Tb_2_(TCA3)_2_] (**f**) and PSS-[Tb_2_(TCA4)_2_] (**g**). The values are presented as the mean ± SD (n = 3).

A thermo-responsivity of materials can be evaluated and compared on a quantitative level by means of the factor *S*_*I*_, defined by Eq. (2).

2 $$S_{I} = \, \Delta I/\left( {I\Delta T} \right)\cdot{1}00\% \, ,$$

where Δ*I* is the change in the luminescence intensity with the temperature variation Δ*T* and *I* is the luminescence intensity at a given temperature. The *S*_*I*_ values were calculated for PSS-[Tb_2_(TCAn)_2_] (n = 3, 4) colloids and plotted versus temperature in Fig. 3e,f. The values range between 2.3–3.5 (heating) and 1.6–3.0 (cooling) for PSS-[Tb_2_(TCA3)_2_], and between 4.8–6.2 (heating) and 2.2–4.6 (cooling) for PSS-[Tb_2_(TCA4)_2_]. These numbers for *S*_*I*_ are higher than the ones reported for inorganic lanthanide-based nanoparticles, being similar to the best ever reported examples and the highest ever reported temperature sensitivity for intracellular temperature nanosensors^30,31^. It is worth noting that the *S*_*I*_-values presented in Fig. 3e,f tend to increase going from dibromo-substituted (TCA3) to tetra-bromo-substituted ligand (TCA4). However, the greater sensitivity of PSS-[Tb_2_(TCA4)_2_] results from the contribution of the photo-induced complex transformations. It is worth assuming that high activity of the complexes at the nanoparticle/water interface is the reason for transformations of the complexes under their heating and prolonged irradiation. Nevertheless, these transformations result in the insignificant release of toxic for living cells Tb^3+^ ions, which can be regarded as a prerequisite of the use of PSS-[Tb_2_(TCAn)_2_] as intracellular detectors of temperature changes. Moreover, the extent of the luminescence reversibility after the heating–cooling cycle for PSS-[Tb_2_(TCA3)_2_] seems to be promising for the reproducibility of the thermally-driven luminescence changes. The latter is important for monitoring of the intracellular temperature changes within several heating/cooling cycles under a cell treatment by hyperthermia. Taking into account that the temperature range can be reduced to 293–318 K to fit into the physiological temperature interval, the reusability of PSS-[Tb_2_(TCA3)_2_] was evaluated in the narrower temperature range (Fig. 4).

The data plotted in Fig. 4a illustrates the quenching and partial recovery of the Tb^3+^-luminescence under the heating and cooling of the aqueous dispersions performed within five heating/cooling cycles. The graph in Fig. 4b represented by I_n_/I_0_ and I_n_/I_(n−1)_ values versus the number of cycles (I_0_, I_n_ and I_(n−1)_ designate the luminescence intensities of the initial colloids, and those at the beginning of n- and (n−1)-cycles, respectively) illustrates smaller degradation of the luminescence after the first cycle. This reveals the possibility to run five heating–cooling cycles at least. Moreover, the *S*_*I*_-values calculated for the same temperature conditions (308 K) remain practically unchanged under the running of the cycles (Fig. 4c). It is also worth noting that the revealed decrease in the luminescence derives in some extent from a transformation of the complex, since the release of toxic Tb^3+^ ions after the heating/cooling cycle is insignificant. Thus, PSS-[Tb_2_(TCA3)_2_] colloids can be considered as cellular nanosensors convenient for recurrent monitoring of the temperature changes, while PSS-[Tb_2_(TCA4)_2_] nanoparticles with the greater thermo-sensitivity than PSS-[Tb_2_(TCA3)_2_] can be applied for single intracellular measurement. Both cytotoxicity and cell internalization of the colloids should be measured before their intracellular application.

### Cytotoxicity, cellular uptake and cellular contrasting ability of PSS-[Tb_2_(TCAn)_2_] (n = 3, 4)

The presentation of the cellular uptake and cytotoxicity of PSS-[Tb_2_(TCAn)_2_] is worth preceding by the discussion of the previously reported data on the PSS-coated colloids based on different lanthanide complexes^37^. It has been, in particular, already estimated that the PSS-based exterior layer of the lanthanide complexes deposited onto the hard cores is a reason for greater cell internalization versus the same hard cores deposited by PSS-polyethyleneimine (PEI) bilayer, where the PEI-layer is the exterior^37^. Moreover, the previously published colloids exhibit rather low cytotoxicity, which makes them efficient cellular contrast agents in confocal microscopy imaging of cell samples^37^. Thus, cell viability measurements of the three cell lines incubated by the colloids has been performed. The results are presented in Fig. 4f,g.

It is worth noting that both colloids exhibit rather low cytotoxicity, although some difference in the cytotoxic effect of PSS-[Tb_2_(TCA3)_2_] and PSS-[Tb_2_(TCA4)_2_] is observed. In particular, the IC_50_ values of PSS-[Tb_2_(TCA3)_2_] evaluated for M-Hela, Chang liver and HSF cell lines are 380, 300 and 340 µM respectively, while the same values for PSS-[Tb_2_(TCA4)_2_] are above 380 µM.

The flow cytometry measurements (Fig. 4d,e) performed for M-Hela cells incubated by PSS-[Tb_2_(TCAn)_2_] (n = 3, 4) colloids under various incubation time indicate the cell internalization of the colloids. Moreover, the data reveal the gradual growth of the cell internalization (derivative of measured luminescence intensity) under the increased incubation time from one to eight hours. The fluorescence microscopy images (Figs. S5,S6) illustrate the time-dependent penetration of the test nanoparticles (PSS-[Tb_2_(TCAn)_2_] (n−3, 4)) into M-Hela cells.

**Figure 5:**
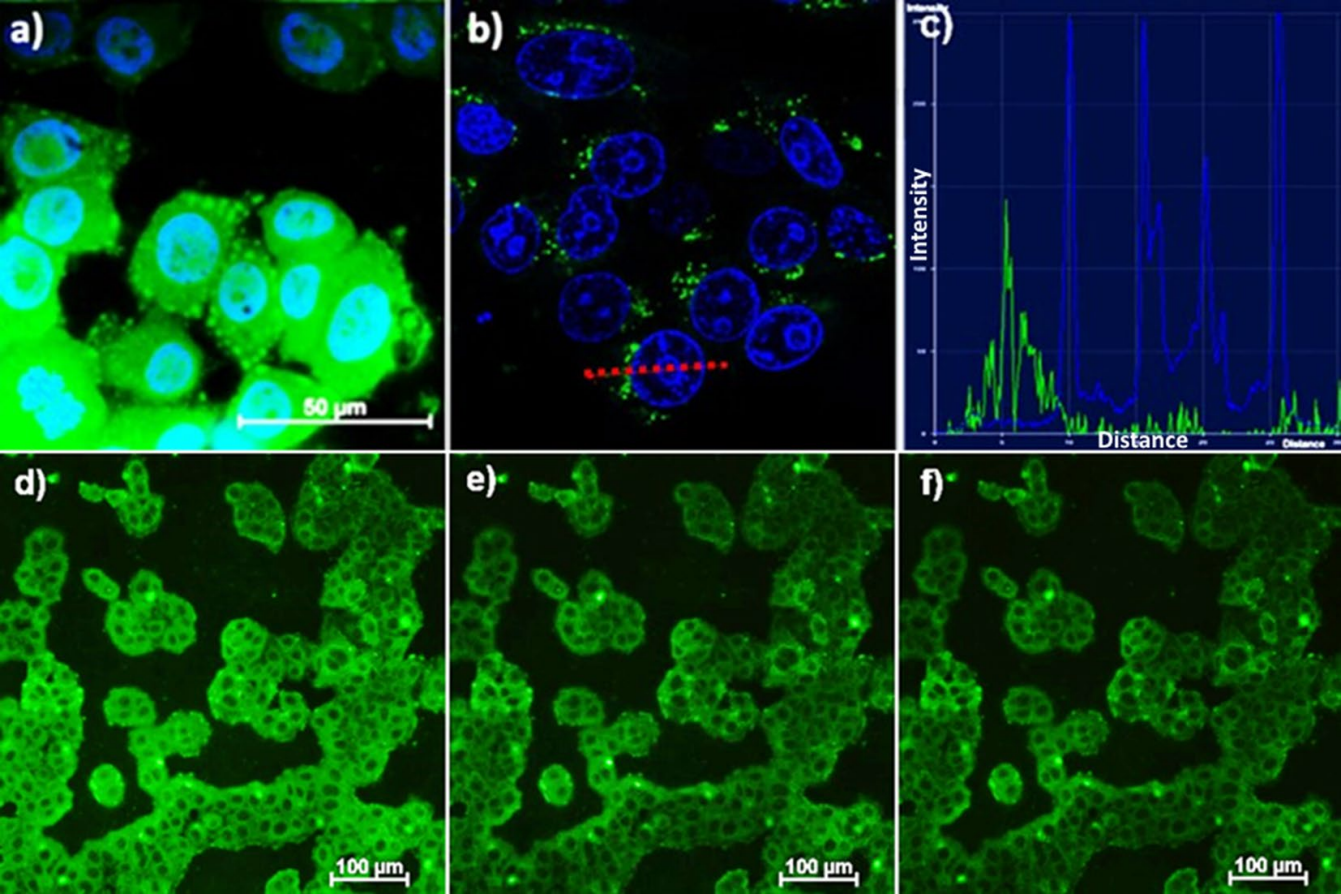
Fluorescent microscopy (**a**) and confocal microscopy (**b**) images of M-Hela cells incubated with PSS-[Tb_2_(TCA4)_2_]. The cell nuclei are stained by DAPI (a) and Hoechst 33,342 (**b**). The graph of luminescence intensity of Hoechst 33,342 (blue) and nanoparticles (green) (**c**) between two points, designated on (**b**) with red line, demonstrating the nanoparticles location outside the cell’s nucleus. Fluorescent microscopy images of M-Hela stained by PSS-[Tb_2_(TCA3)_2_] at different temperatures (**d**) 30 °C, (**e**) 35 °C, (**f**) 40 °C.

The internalization of PSS-[Tb_2_(TCA3)_2_] and PSS-[Tb_2_(TCA4)_2_] into M-Hela cells was visualized by both fluorescence microscopy and confocal images of the cell samples incubated by the colloids. Figure 5a shows the images obtained by fluorescence microscopy for the cell samples incubated for 8 h by PSS-[Tb_2_(TCA4)_2_] and confocal microscopy for the cell samples incubated for 24 h. No detectable difference between the colloids is revealed in their cell internalization. It is worth noting that the confocal image with the staining of the cell nuclei by the dye (blue spots in Fig. 5b) details the location of PSS-[Tb_2_(TCA4)_2_] within cell cytoplasm. Moreover, Fig. 5c indicates that the nanoparticles are in close proximity to the cell nuclei, but do not enter them. The efficient cell internalization of the PSS-coated colloids seems to disagree with their high negative surface charge manifested by the ζ-values in Table 1. However, interactions of nanoparticles with proteins present in the cell culture commonly results in a formation of protein corona. Similar tendency has been previously reported for the PSS-coated colloids based on the lanthanide complexes with 1,3-diketonate derivative of calix[4]resorcinarene^51^. The protein-corona, in turn, is the well-known factor modifying the cell internalization of different types of nanoparticles^52^, which has been previously exemplified for the silica nanoparticles bearing sulfonate-groups at their surface^53^. Thus, efficient cell internalization of the PSS-coated colloids manifested by the marking of the cell cytoplasm provides a basis for their use as intracellular transducers of the temperature changes.

The ability of the nanoparticles to monitor the temperature changes was experimentally demonstrated by fluorescent microscopy measurements of the cell sample preliminary incubated by the nanoparticles both before and after the heating by means of the thermostat with the temperature control, which is illustrated by the Scheme in the Experimental section. The images of the cell samples made at different temperatures are reported in Fig. 5d–f. The temperature-induced quenching in the temperature range 303–313 K is evident by a naked eye, while the evaluation of the mean intensity at 546 nm provides quantitative confirmation of the trend (Table 3).

**Table 3:** Mean intensity values at 546 nm measured by fluorescent microscopy at different temperatures.

Image	5d)	5e)	5f)
Temperature, °C	30	35C	40
Arithmetic mean intensity	27,03	20,42	17,93

## Conclusions

The work introduces synthesis of hydrophilic core–shell nanoparticles exhibiting thermo-responsive Tb^3+^-centered luminescence that is dependent on the structure of the ligands. The latter are both unsubstituted and upper-rim substituted thiacalix[4]arenes with four *tert*-buthyl-, two or four bromo-substituents. The synthesis was based on solvent-induced nanoprecipitation of the Tb^3+^ complexes, while a hydrophilic coating of the cores by polystyrenesulfonate (PSS) molecules provides their colloid stability. The hydration number equal to 2 was evaluated by the time-resolved luminescence measurements for the nanoprecipitated complexes with the unsubstituted, *tert*-buthyl-substituted and dibromo-substituted ligands. This indicates that specific dimeric complexes with sandwich-like coordination mode of Tb^3+^ ions, which are predominant in the DMF solutions, remain unchanged after the nanoprecipitation. The greater (close to 3) hydration number obtained for the complexes with tetra-brominated thiacalix[4]arene can be explained by a contribution of the monomeric complexes with the greater hydration number of Tb^3+^ ions. The bromo-substitution of thiacalix[4]arene causes the quenching of the Tb^3+^-centered luminescence due to the energy back transfer, which increases with the heating in the temperature range 293–333 K. The deviation of the luminescence intensities measured under the heating and cooling of the colloids indicates that the temperature-dependent luminescence of the colloids has a contribution coming from the irreversible transformation of the complexes. Thus, the greatest irreversible contribution to the heating-induced quenching of the luminescence makes the nanoparticles based on the complexes with tetra-brominated thiacalix[4]arene the most sensitive to the heating, but restricts their applicability to the single use only. However, the di-brominated ligands provide the optimal ligand environment of the Tb^3+^ ions in the nanoparticles for the recurrent temperature measurements for five heating/cooling cycles at least. The heating/cooling cycle results in the insignificant release of toxic Tb^3+^ ions from the nanoprecipitated complexes with the di-brominated thiacalix[4]arenes. This along with the low cytotoxicity of the PSS-coated colloids estimated for the three cell lines points to their applicability as intracellular sensors of temperature changes. The quick cell internalization of the colloids results in the efficient marking of the cell cytoplasm by green luminescence. The latter exhibit detectable quenching under the heating of the cell samples in the temperature range within 303–313 K. This study demonstrates the successful application of complex colloidal structures for monitoring the intracellular temperature changes with unprecedented sensitivity, and may represent a significant step forward for accurate 2D temperature mapping in processes like hyperthermia or pathological processes.

## Supplementary information

Supplementary Information.

## Data Availability

All data generated or analyzed during this study are included in this published article (and its Supplementary Information files).
