# Supplementary material for: Terbium(III)-thiacalix[4]arene nanosensor for highly sensitive intracellular monitoring of temperature changes within the 303–313 K range
Source: Sci Rep. 2020 Nov 25;10:20541. doi: 10.1038/s41598-020-77512-1 (PMC7689473; doi:10.1038/s41598-020-77512-1)
Supplement: Supplementary file 1 — Supplementary Information. [file 41598_2020_77512_MOESM1_ESM.doc]

**Supplementary Material**

**Terbium(III)-thiacalix[4]arene nanosensor for highly sensitive intracellular monitoring of temperature changes within the 303-313 K range**

Rustem Zairov,*^a^ Alexey P. Dovzhenko,^b^ Anastasiia S. Sapunova^a^, Alexandra D. Voloshina^a^, Kirill Sarkanich,^b^ Amina Daminova,^b^ Irek Nizameev,^a^ Dmitry Lapaev,^c^ Svetlana Sudakova,^a^ Sergey N. Podyachev,^a^ Konstantin A. Petrov^a^, Alberto Vomiero*^d,e^ and Asiya R. Mustafina^a^

^a^Arbuzov Institute of Organic and Physical Chemistry, FRC Kazan Scientific Center, Russian Academy of Sciences, 8 Arbuzov str., 420088 Kazan, Russian Federation.

^b^Kazan (Volga region) Federal University, 18 Kremlyovskaya str., 420008 Kazan, Russian Federation.

^c^ Zavoisky Physical-Technical Institute, FRC Kazan Scientific Center of RAS, Sibirsky tract, 10/7, 420029, Kazan, Russian Federation.

^d^ Division of Materials Science, Department of Engineering Sciences and Mathematics, Luleå University of Technology, SE-971 87 Luleå, Sweden.

^e^ Department of Molecular Sciences and Nanosystems, Ca’ Foscari University of Venice, via Torino 155, 30172 Venezia-Mestre, Italy.

# Experimental Section

*Dynamic light scattering (DLS)* measurements were performed using Malvern Mastersize 2000 particle analyzer operating with a He–Ne laser (633 nm) and emitting vertically polarized light as a light source. *Transmission electron microscopy (TEM)* images have been obtained with 120 (Carl Zeiss), Japan. Samples have been sonicated in water for 30 min and then dispersed on 200 mesh copper grids with continuous formvar support films. The images have been acquired at an accelerating voltage of 100 kV. *Luminescence spectra and lifetime measurements* in visible range have been recorded using Hitachi F-7100 luminescent spectrometer. The measurements were performed at different temperatures in aerated conditions with the use of Peltier system for the temperature control.

The time-resolved luminescence spectra of the Gd^3+^ complexes were recorded using an optical spectrometer based on an MDR-23 grating monochromator (LOMO, Saint Petersburg, Russia) coupled to a FEU-100 photomultiplier tube.^45^ The luminescence was excited by an LGI-21 pulsed nitrogen laser (337 nm wavelength, 2.1 mW laser pulse average output power, 10 ns pulse duration, 100 Hz repetition rate). The average output power of the laser near the samples was 1.7 mW. The exposed surface areas of the samples were 7 mm^2^.

*Transmission electron microscopy (TEM)* images have been obtained with 120 (Carl Zeiss), Japan. Samples have been sonicated in water for 30 min and then dispersed on 200 mesh copper grids with continuous formvar support films. The images have been acquired at an accelerating voltage of 100 kV. EDS experiment was carried out on transmission electron microscope using energy-dispersive X-ray detector from Thermo Scientific.

## Cytotoxicity assays

Cytotoxic effects of the test compounds on human line cells were estimated by means of the multifunctional Cytell Cell Imaging system (GE Health Care Life Science, Sweden) using the Cell Viability Bio App which precisely counts the number of cells and evaluates their viability from fluorescence intensity data. Two fluorescent dyes that selectively penetrate the cell membranes and fluoresce at different wavelengths were used in the experiments. A low-molecular-weight 4′,6-diamidin-2-phenylindol dye (DAPI) is able to penetrate intact membranes of living cells and color nuclei in blue. High-molecular propidium iodide dye penetrates only dead cells with damaged membranes, staining them in yellow. As a result, living cells are painted in blue and dead cells are painted in yellow. DAPI and propidium iodide were purchased from Sigma. The M-Hela clone 11 human, epithelioid cervical carcinoma, strain of Hela, clone of M–Hela; from the Type Culture Collection of the Institute of Cytology (Russian Academy of Sciences) and Chang liver cell line (Human liver cells) from N. F. Gamaleya Research Center of Epidemiology and Microbiology and Fibroblast-epithelial models isolated from a conditionally healthy donor were used in the experiments (from Kazan Federal University collection, Kazan, Russia). The cells were cultured in a standard Eagle’s nutrient medium manufactured at the Chumakov Institute of Poliomyelitis and Virus Encephalitis (PanEco company) and supplemented with 10% fetal calf serum and 1% nonessential amino acids. The cells were plated into a 96-well plate (Eppendorf) at a concentration of 1×10^5^ cells/mL, 150 μL of medium per well, and cultured in a CO_2_ incubator at 37°C. Twenty four hours after seeding the cells into wells, the compound under study was added at a preset dilution, 150 μL to each well. The dilutions of the compounds were prepared immediately in nutrient media. The experiments were repeated three times. Intact cells cultured in parallel with experimental cells were used as a control.

## Statistical analysis.

The cytometric results were analyzed by the Cytell Cell Imaging multifunctional system using the Cell Viability BioApp. The data in the tables and graphs are given as the mean ± standard error.

## Flow cytometry assay.

Fluorescence microscopy. After treatment with the PSS-[Tb_2_(TCA3)_2_] and PSS-[Tb_2_(TCA4)_2_], M-Hela cells were fixed and stained with DAPI (blue). The survey was carried out using a Nikon Eclipse Ci-S fluorescence microscope (Nikon, Japan) at a magnification of 1000x for oil immersion objective and 400x.

## Confocal laser scanning microscopy.

The cells were grown on glasses in microbiological tablet «Eppendorf» for 24 h at 37 ºC in CO_2_-incubator. After the addition of PSS-[Tb_2_(TCA3)_2_] with M-Hela cell line during 24 h the samples were stained using Hoechst 33342 (blue fluorescent) according to the manufacturer’s instructions. The nanoparticles were visualized with inverted confocal laser scanning microscope (LSM 780, Carl Zeiss, Germany).

*Determination of the T_1_ state energy of the ligand TCA2 in its Gd^3+^ complex*

The phosphorescence spectra of the Gd^3+^ complexes TCA2 at 146 K and 40 µs time delay are characterized by broad bands with maxima at 417 nm (23981 cm^-1^) (Figure S2e). Since this spectrum do not contain a fine vibrational structure, required to accurate determination of the position of the maximum of the 0–0 phonon transition corresponding to the energy of the T_1_ of the state of the ligand, we used a spectral deconvolution of the spectra into a series of overlapping bands using a Gaussian function [S. Shuvaev, V. Utochnikova, Ł. Marciniak, A. Freidzon, I. Sinev, R. Van Deun, R.O. Freire, Y. Zubavichus, W. Grünert and N. Kuzmina, Dalton Trans., 2014, **43**, 3121-3136; Xian-Sheng Ke, Bo-Yan Yang, Xin Cheng, Sharon Lai-Fung Chan, and Jun-Long Zhang, Chem. Eur. J. 20 (2014) 4324-4333]. Energy value of the *T*_1_ states of the ligands were determined from the maxima of short-wavelength deconvoluted bands. The value for the Gd^3+^ complexes TCA2 (417 nm (23981 cm^-1^), respectively.

## Table S1 Tb^3+^ losses without and after the heating.

| PSS-[Tb_2_(TCAn)_2_] | C(initial), mM | Synthesis losses, % | Washing losses, % | Losses after heating (293-323 K), % |
| --- | --- | --- | --- | --- |
| n=1 | 0.75 | 3.04 | 1.78 | 1.55 |
| n=2 |  | 3.32 | 0.23 | 1.88 |
| n=3 |  | 3.26 | 0.39 | 0.12 |
| n=4 |  | 3.62 | 0.91 | 0.27 |

## Table S2 Excited state lifetimes (τ1 and 2) for PSS-[Tb2(TCAn)2] and corresponding hydration numbers (q).

| PSS-[Tb_2_(TCAn)_2_] | | A_1_ | τ_1_ | A_2_ | τ_2_ | Adj. R-Square | τ_avg_ | q |
| --- | --- | --- | --- | --- | --- | --- | --- | --- |
| n=1 | H_2_O | 1429.74 | 0.789 | 2806.70 | 1.25 | 0.99999 | 1.14 | 1.65 |
|  | D_2_O | 4815.92 | 2.28 | 2196.14 | 1.04 | 0.99999 | 2.07 |  |
| n=2 | H_2_O | 3143.33 | 0.84 | 1478.50 | 0.41 | 0.99993 | 0.76 | 2.15 |
|  | D_2_O | 3068.36 | 0.62 | 4030.55 | 1.44 | 0.99995 | 1.24 |  |
| n=3 | H_2_O | 1272.34 | 0.85 | 604.96 | 0.42 | 0.99998 | 0.77 | 1.93 |
|  | D_2_O | 1234.26 | 1.44 | 1253.03 | 0.63 | 0.99998 | 1.19 |  |
| n=4 | H_2_O | 477.51 | 0.31 | 344.44 | 0.60 | 0.99933 | 0.48 | 3.01 |
|  | D_2_O | 1046.40 | 0.96 | 1833.92 | 0.44 | 0.99993 | 0.73 |  |

## Table S3 DLS data after heating-cooling cycle.

| PSS-[Tb_2_(TCAn)_2_] | Hydrodynamic parameters before temperature tests | | | Hydrodynamic parameters after temperature tests | | |
| --- | --- | --- | --- | --- | --- | --- |
|  | **d, nm** | **PDI** | **ζ, mV** | **d, nm** | **PDI** | **ζ, mV** |
| n=1 | 100.1 | 0.217 | -59.3 | 100.3 | 0.171 | -45.6 |
| n=2 | 124.5 | 0.124 | -45.8 | 97.56 | 0.319 | -37.8 |
| n=3 | 82.75 | 0.239 | -67.7 | 129.0 | 0.273 | -46.8 |
| n=4 | 81,17 | 0,191 | -57,4 | 79.6 | 0.255 | -41.6 |

Hydrodynamic diameter (d) measurement error do not exceed 5 nm, PDI measurement error do not exceed 0.02, electrokinetic potential (**ζ**) measurement error do not exceed 2 mV.

# Supplementary figures

a*
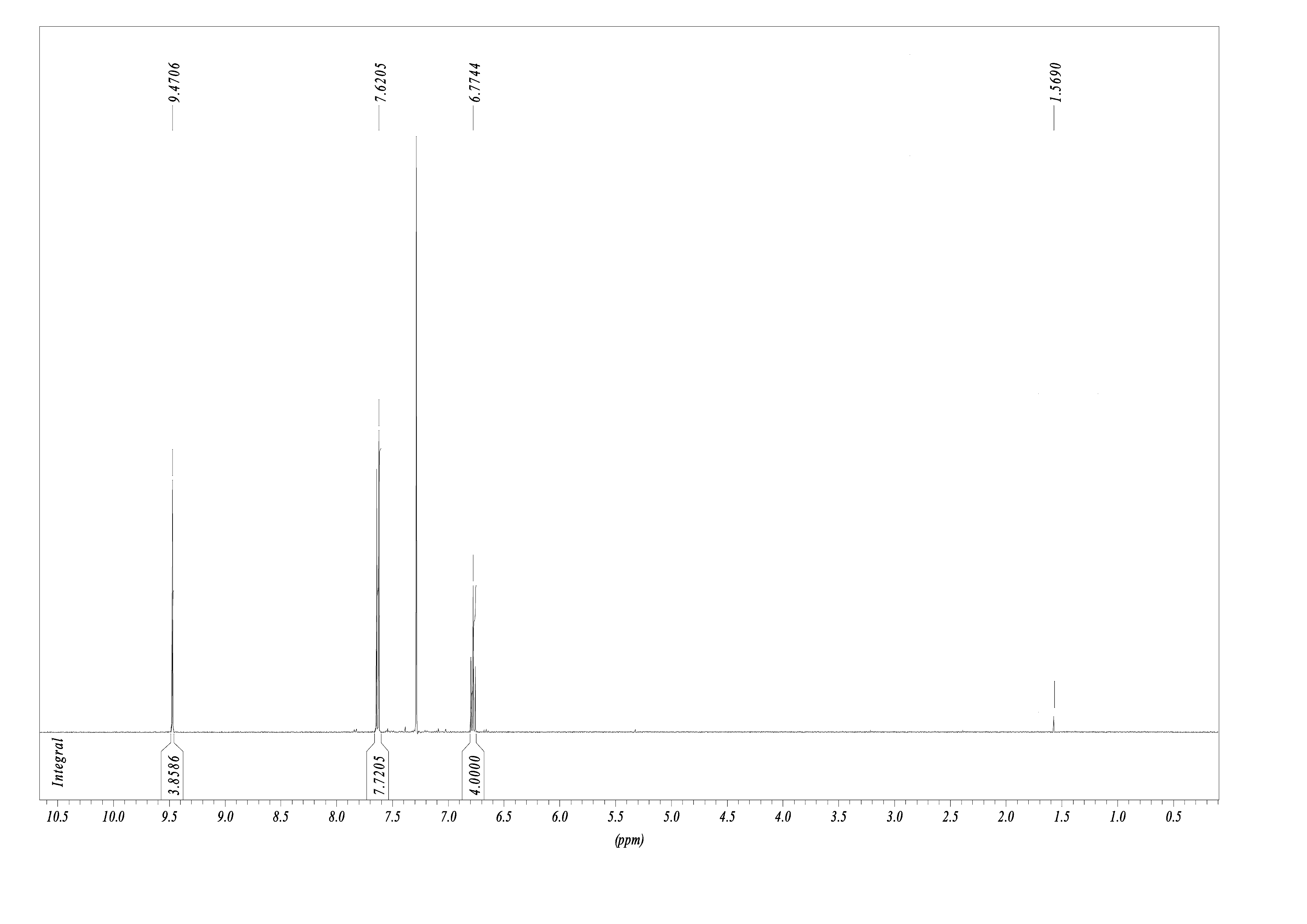
*

b
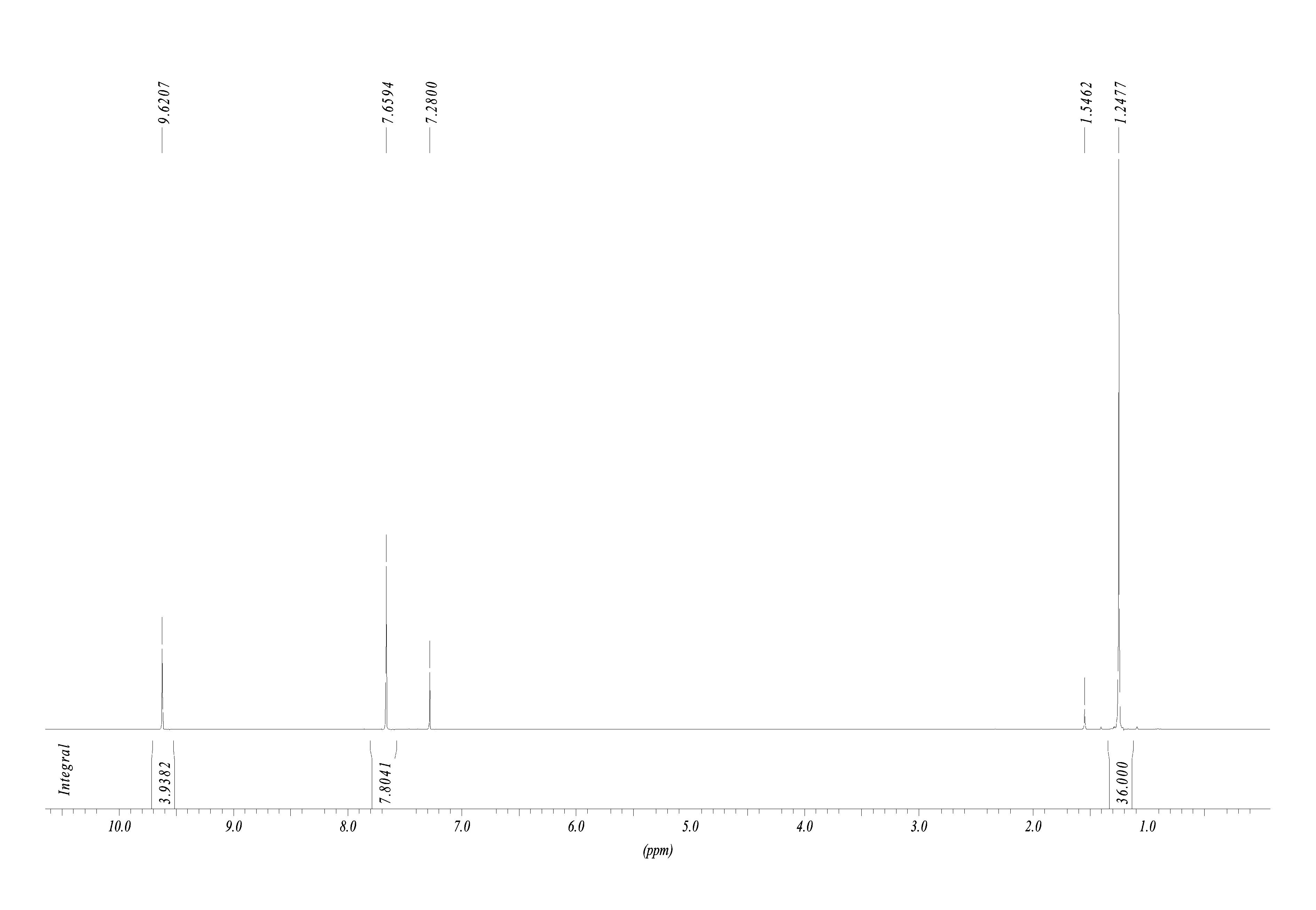


c*
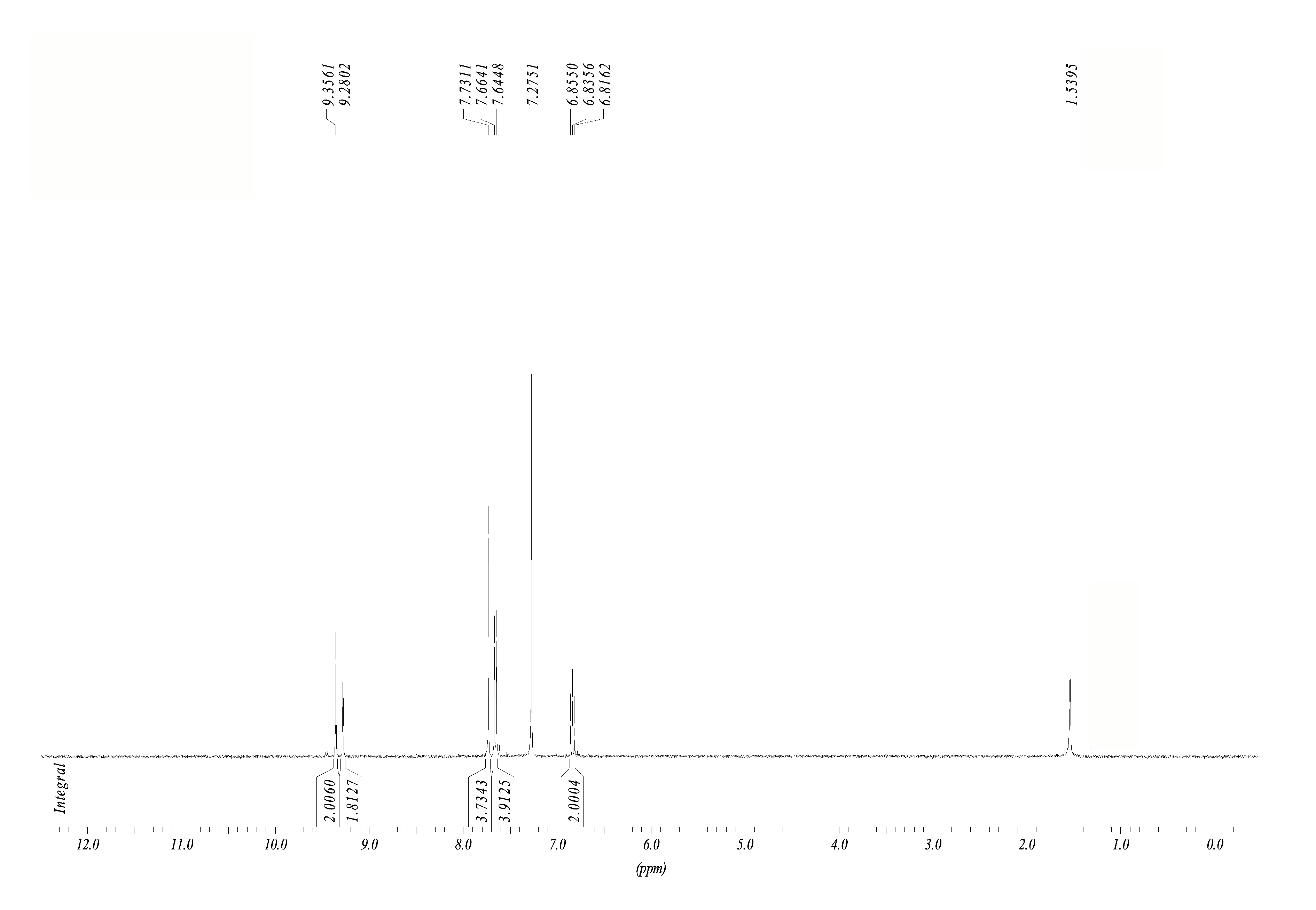
*

d*
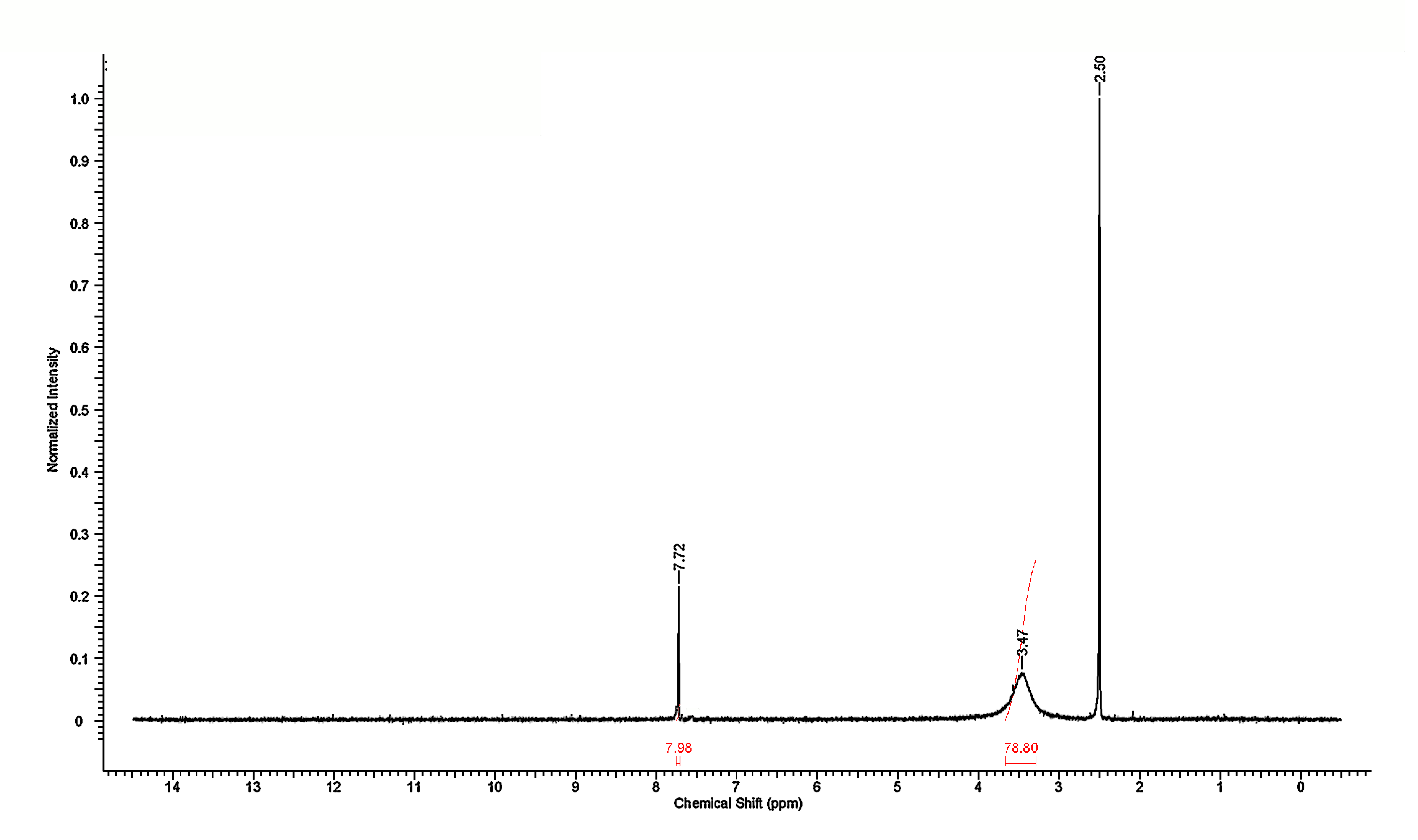
*

**Figure S1.** ^1^H NMR spectra of compounds (a) **TCA1** (CDCl_3_), (b) **TCA2** (CDCl_3_), (c) **TCA3** (CDCl_3_) and (d) **TCA4** (DMSO-d_6_).


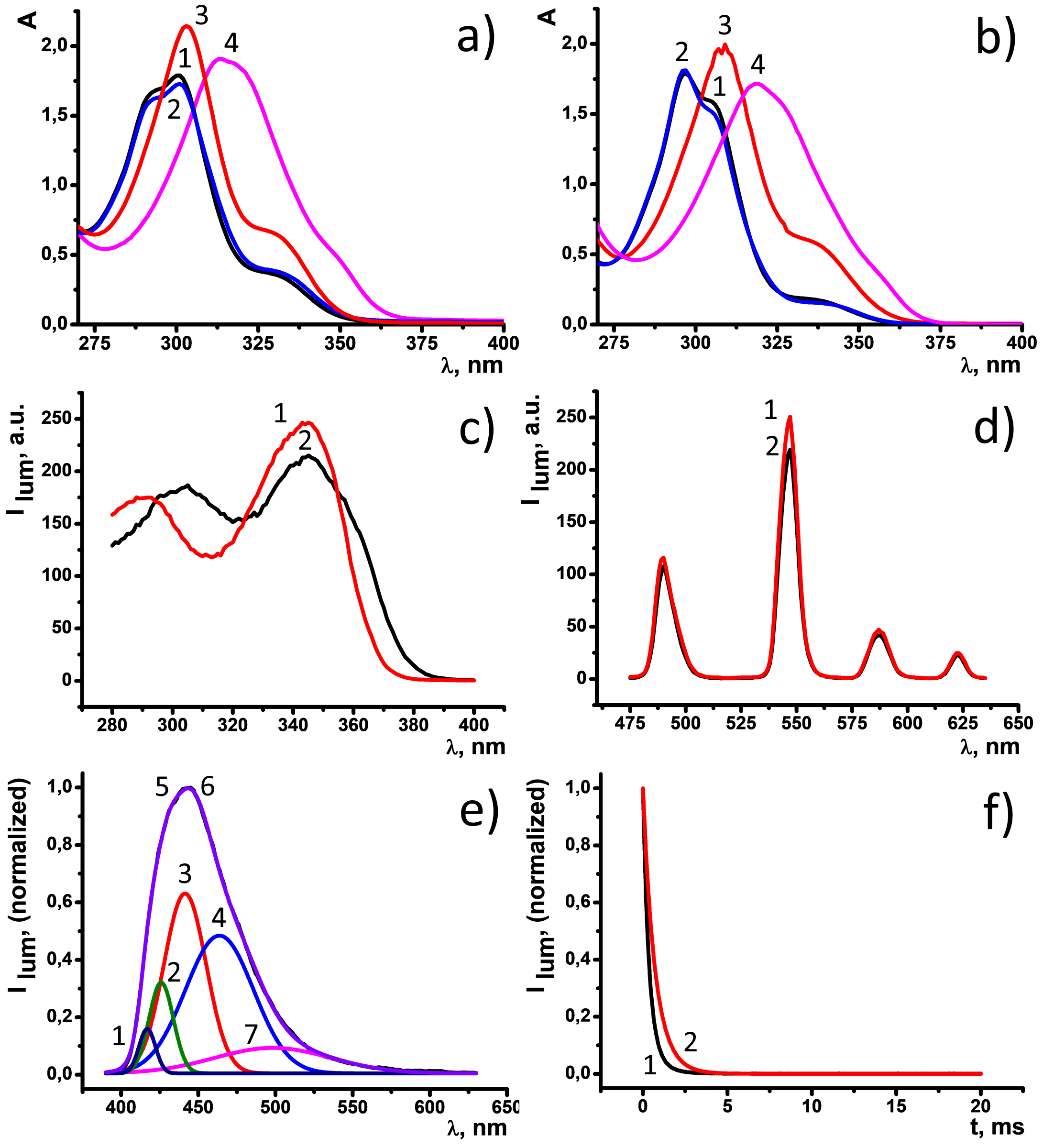


**Figure S2.** UV-Vis absorption spectra of 1 - L (L=TCA1 (a), TCA2 (b)) (C_L_=0.1 mM) in DMF; 2 - L with Tb(NO_3_)_3_ (C_Tb_^3+^ = 0.1 mM) (L-Tb (1:1)); 3 - L with TEA (C_TEA_=0.8 mM) (L-TEA (1:8)); 4 - L with Tb(NO_3_)_3_ (C_Tb_^3+^ = 0.1 mM) and TEA (C_TEA_=0.8 mM) (L-Tb^3+^-TEA (1:1:8)). The excitation (c) and luminescence spectra (d) of the terbium complexes with ligands TCA1 (λ_ex_ = 343 nm) and TCA2 (λ_ex_ = 345 nm) in alkaline DMF solutions. C_Tb_^3+^ = C_L_ = 0.1 mM, L:Tb^3+^:TEA (1:1:8). (e) A spectral deconvolution of the normalized phosphorescence spectra of the Gd^3+^ complexes TCA2 at 40 µs time delay and T = 146 K into subcomponent by using a Gaussian curve fitting. (f) Luminescence decay curves of PSS-[Tb_2_(TCA1)_2_] (1) and PSS-[Tb_2_(TCA2)_2_] (2).


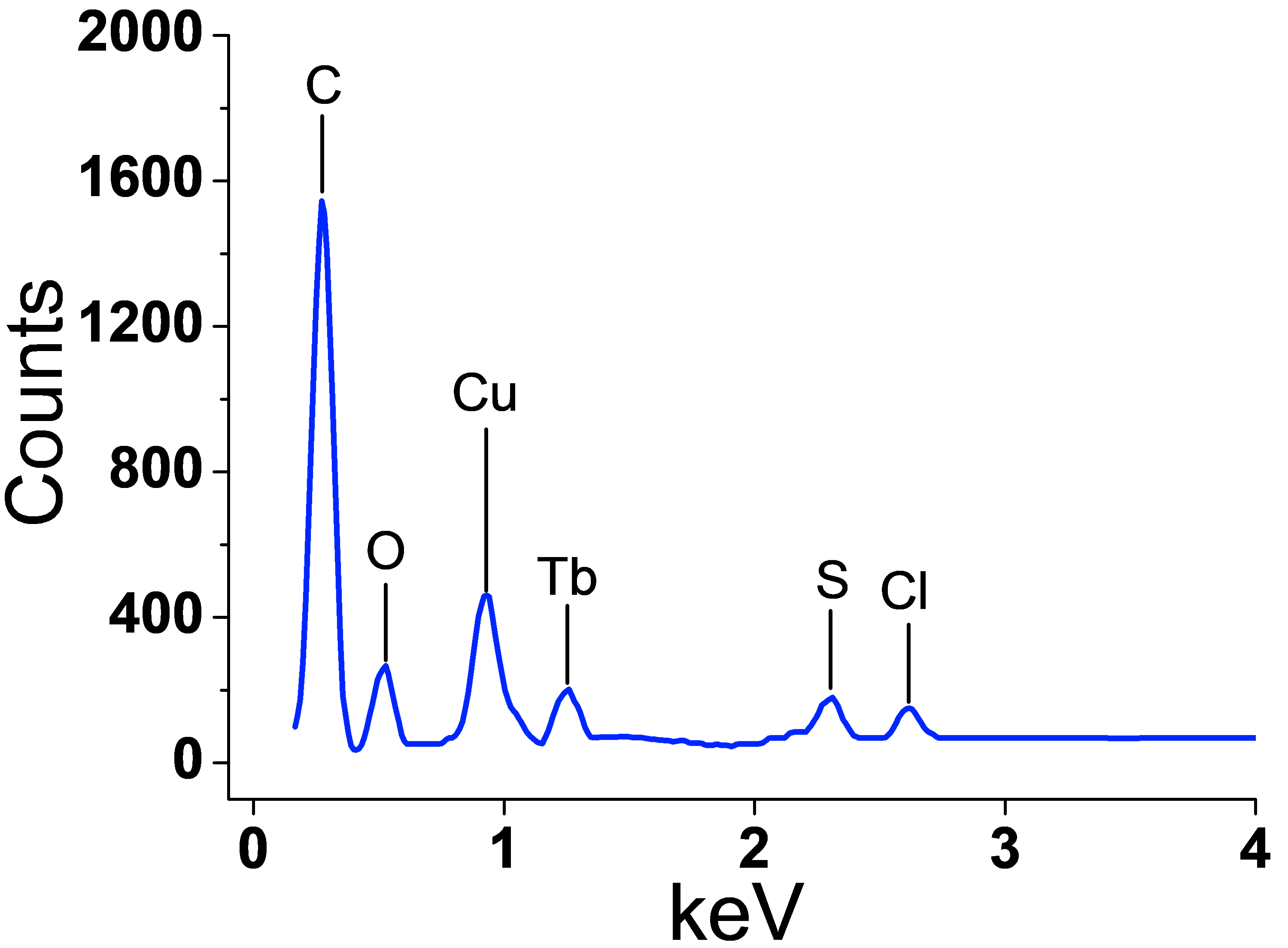


Figure S3. EDS spectrum of PSS-[Tb_2_(TCA3)_2_].


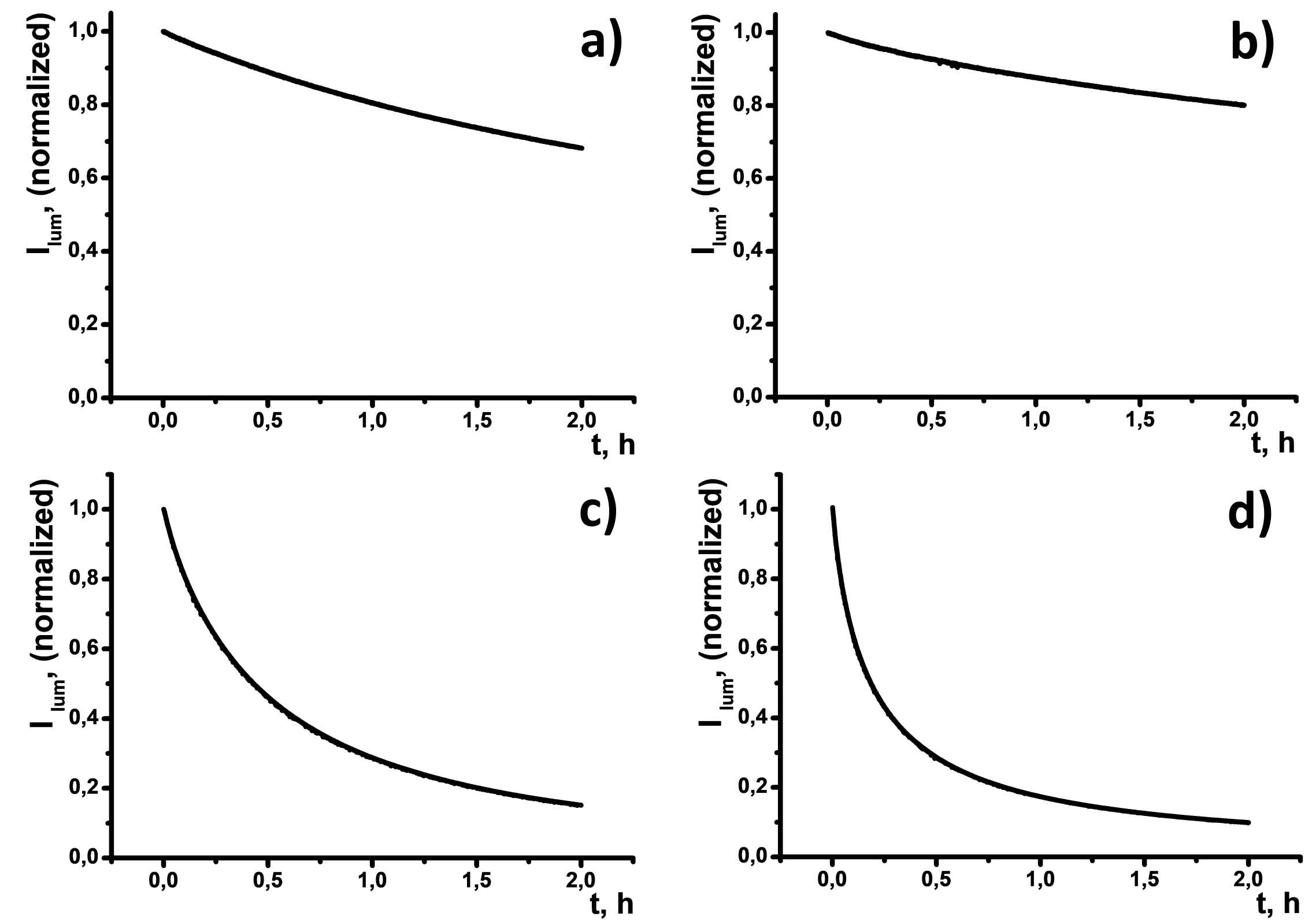


## Figure S4. Photobleaching (I_lum_ versus time) for (a) PSS-[Tb_2_(TCA1)_2_], (b) PSS-[Tb_2_(TCA2)_2_], (c) PSS-[Tb_2_(TCA3)_2_] and (d) PSS-[Tb_2_(TCA4)_2_].

| **Dapi** | PSS-[Tb_2_(TCA3)_2_] | **Merge** | **Time,h** |
| --- | --- | --- | --- |
| 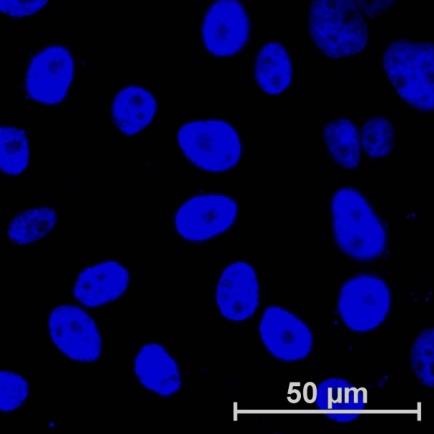 | 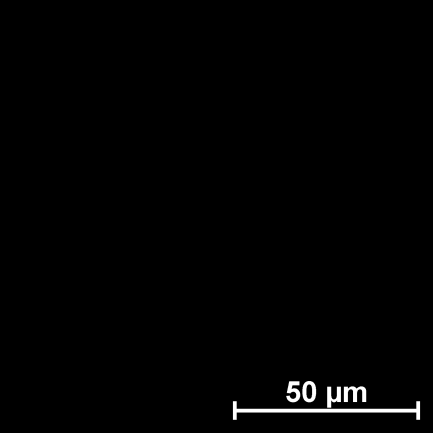 | 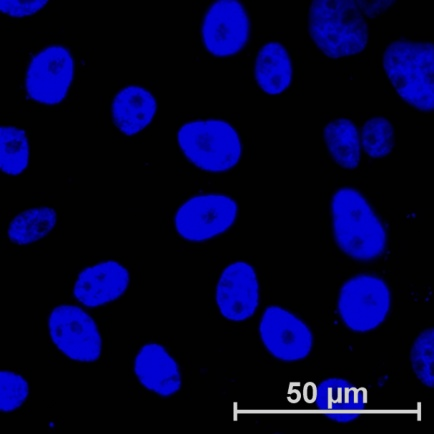 | **Control**  **M-Hela** |
| 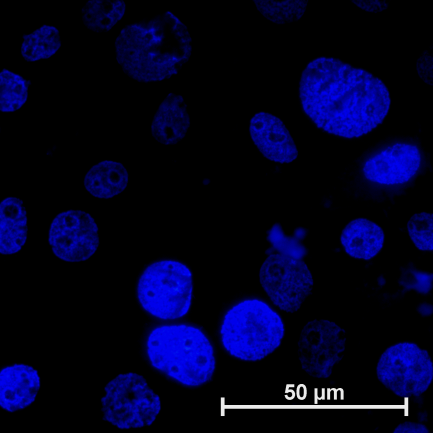 | 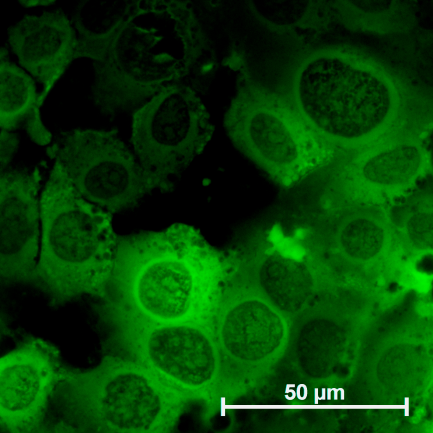 | 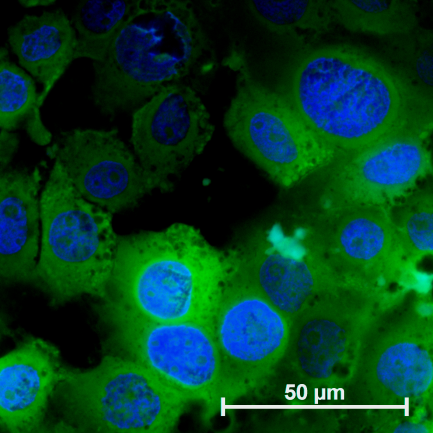 | **2 h** |
| 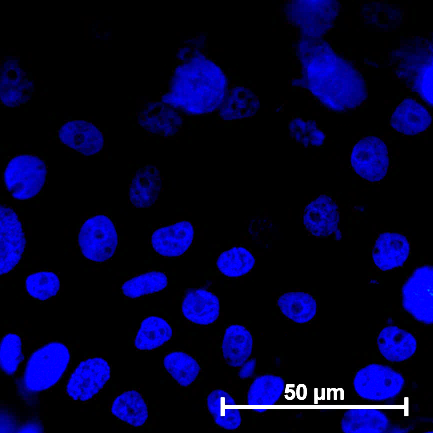 | 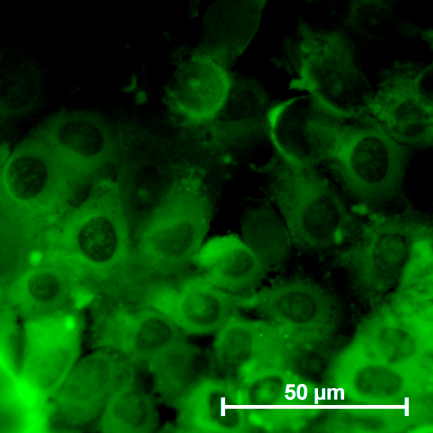 | 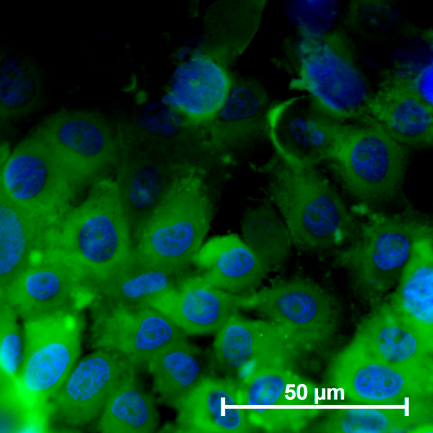 | **4 h** |
| 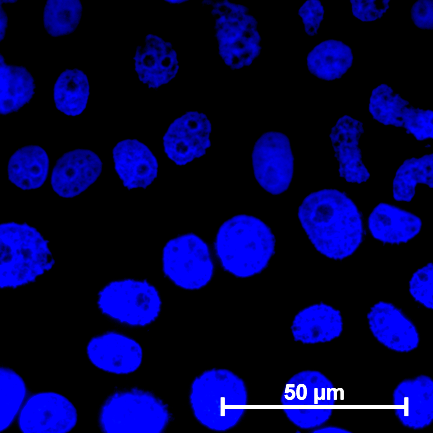 | 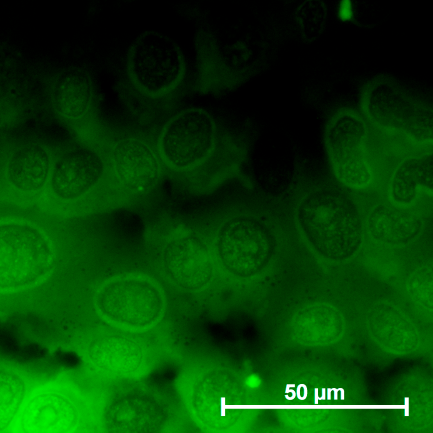 | 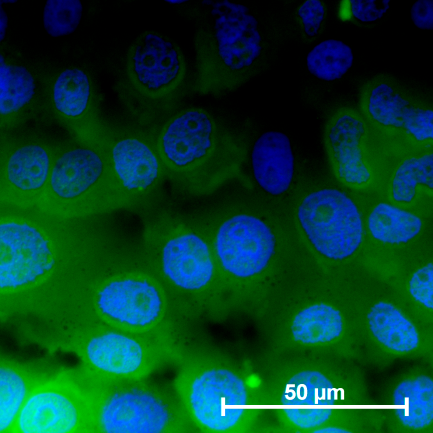 | **8 h** |
| 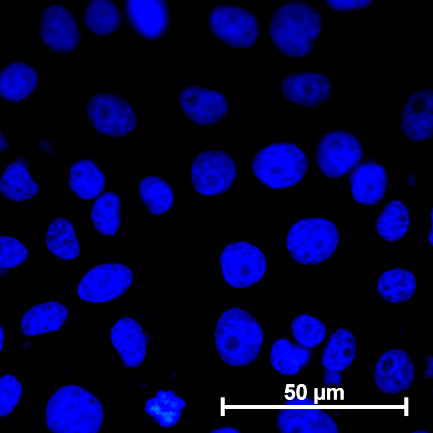 | 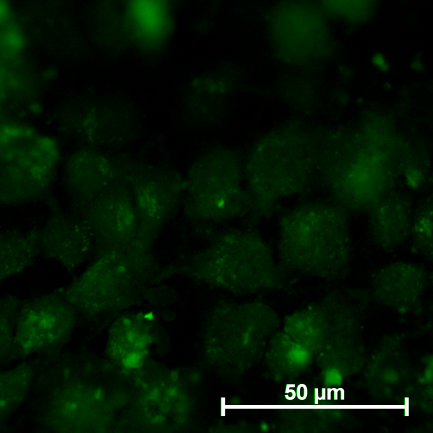 | 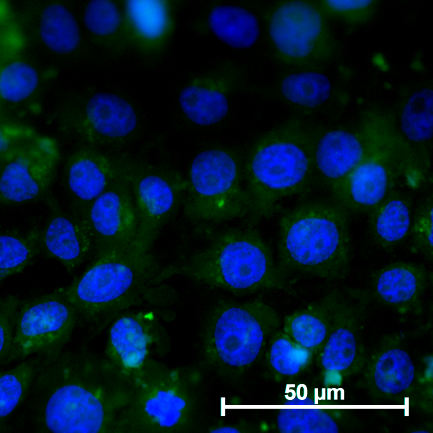 | **24 h** |

## Figure S5. Cellular uptake study of PSS-[Tb_2_(TCA3)_2_] by fluorescence microscopy.

| **Dapi** | **PSS-[Tb_2_(TCA4)_2_]** | **Merge** | **Time, h** |
| --- | --- | --- | --- |
| **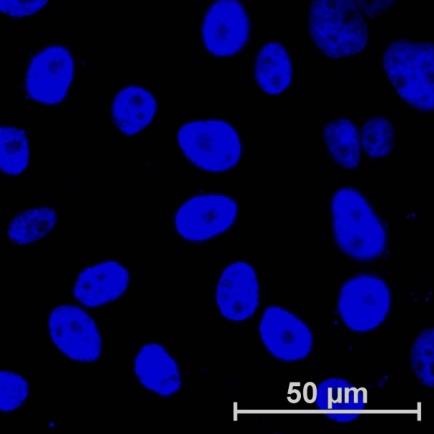** | **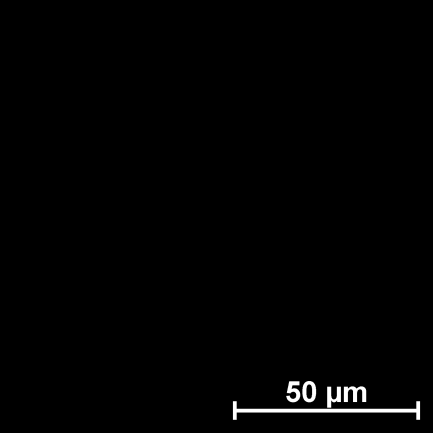** | **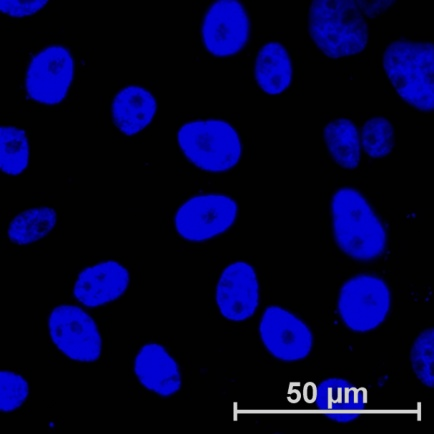** | **M-Hela** |
| **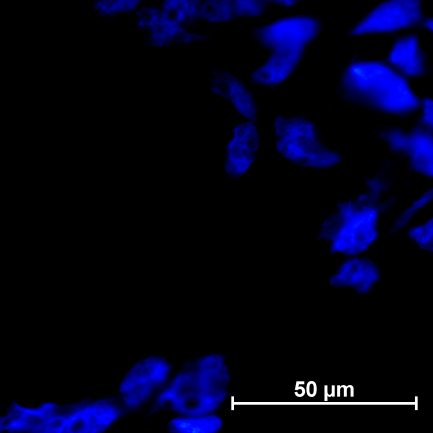** | **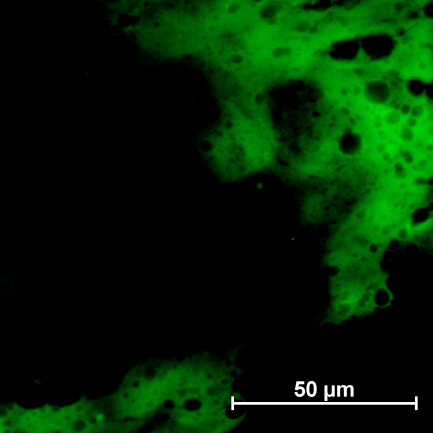** | **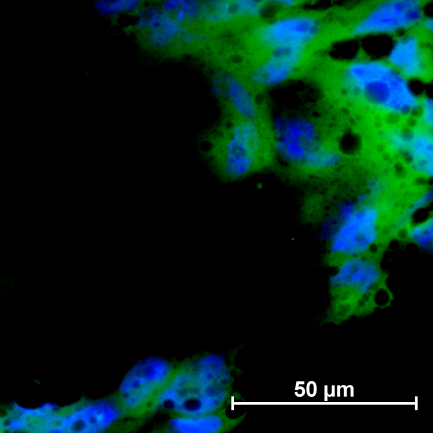** | **2 h** |
| **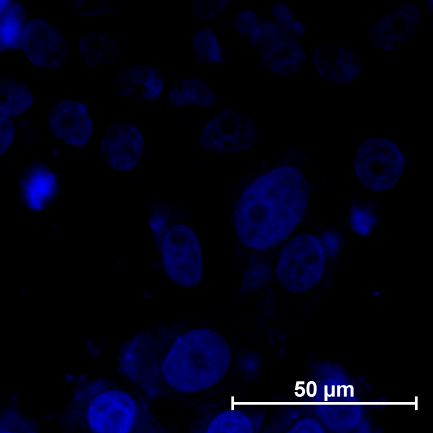** | **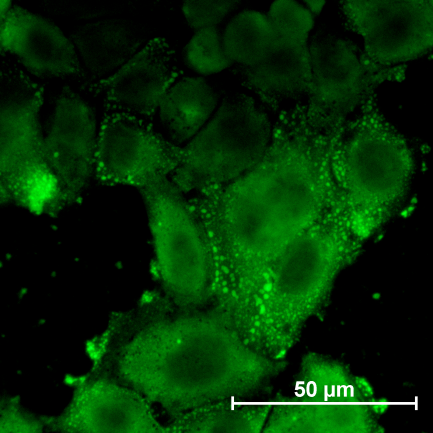** | **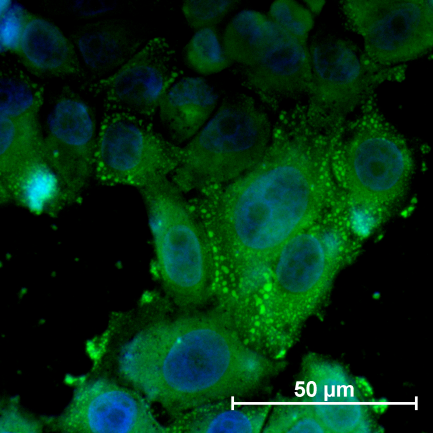** | **4 h** |
| **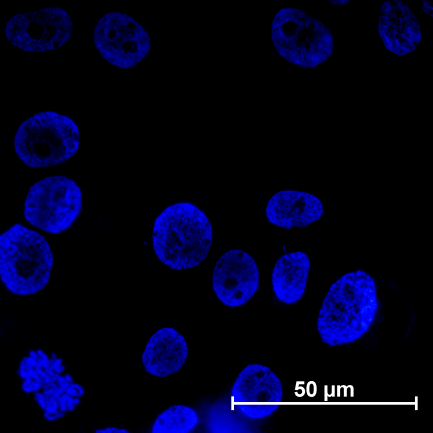** | **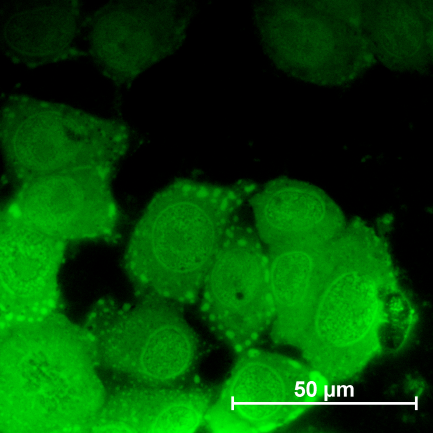** | **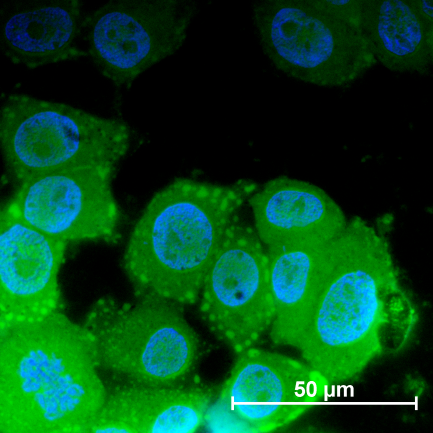** | **8 h** |
| **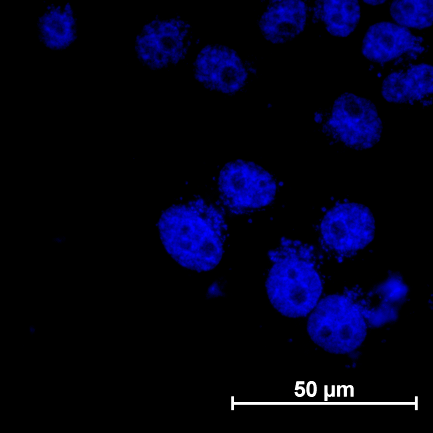** | **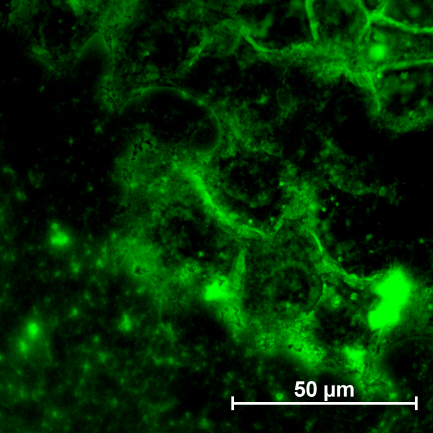** | **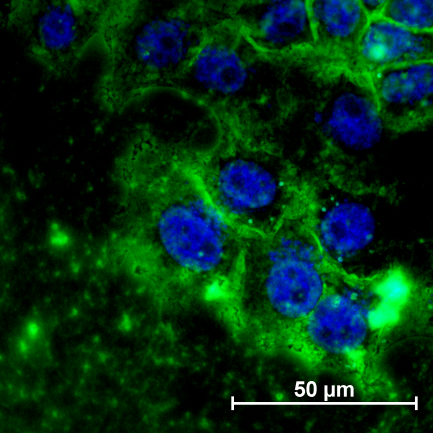** | **24 h** |

## Figure S6. Cellular uptake study of PSS-[Tb_2_(TCA4)_2_] by fluorescence microscopy.
